# Illuminating ecology and distribution of the rare fungus *Phellinidium pouzarii* in the Bavarian Forest National Park

**DOI:** 10.1038/s41598-025-91672-y

**Published:** 2025-03-12

**Authors:** Friederike Roy, Philipp Baumann, René Ullrich, Julia Moll, Claus Bässler, Martin Hofrichter, Harald Kellner

**Affiliations:** 1 Department of Bio- and Environmental Sciences, International Institute Zittau, TU Dresden, Markt 23, 02763 Zittau, Germany; 2 Department for Biology, Chemistry and Geo Sciences, Institute for Ecology of Fungi, University Bayreuth, Universitätsstraße 30, 95447 Bayreuth, Germany; 3 National Park Bavarian Forest, Freyunger Str. 2, 94481 Grafenau, Germany; 4 Department of Soil Ecology, Helmholtz Centre for Environmental Research, Theodor-Lieser- Straße 4, 06120 Halle (Saale), Germany

**Keywords:** Rare fungi, Fungal conservation, Fungal genomics, Biodiversity, Conservation biology, Community ecology, Genomics

## Abstract

Due to their cryptic lifestyle, hidden diversity and a lack of ecological knowledge, conservation of wood-inhabiting fungi continues to be a niche interest. Molecular methods are able to provide deeper insights into the ecology of rare fungal species. We investigated the occurrence of the rare wood-inhabiting fungus *Phellinidium pouzarii* across the Bavarian Forest National Park in Germany using a fruit body survey, amplicon sequencing and qPCR. Additionally, we sequenced the genome of *P. pouzarii* and characterized the chemical substances responsible for its distinctive scent. Our approach gave matching results between amplicon sequencing and qPCRs, however, we found no evidence that *P. pouzarii* is more abundant in the National Park than we can assume based on fruit body inventories, underlining the species’ critically endangered status. Genomics revealed *P. pouzarii*’s repertoire of ligninolytic enzymes, pointing towards a white rot lifestyle. Two main components of *P. pouzarii*’s distinct odour we identified (2-phenylethanol, methyl *p*-anisate) are known to act as insect attractants and/or to possess antimicrobial properties.

**Supplementary Information:**

The online version contains supplementary material available at 10.1038/s41598-025-91672-y.

## Introduction

Despite their major contributions to the functioning of terrestrial ecosystems^1^, fungi have until recently largely been neglected by institutional nature conservation^2–4^. For instance, the list of species protected under the Habitats Directive of the European Union (Council Directive 92/43/EEC) does not contain a single fungal species.

Conservation of wood-inhabiting fungi in particular is inherently difficult due to a lack of knowledge on true diversity, the ecology of single species^5^, the “hidden” lifestyle of many species with low frequency of producing fruit bodies^2,6^ and the often unclear taxonomic resolution^7^. It is therefore difficult to accurately assess the endangerment of a presumably rare fungal species.

Over the last decades, molecular techniques such as Next Generation Sequencing (NGS) or Genomics have offered new insights into the ecology and physiology of single fungal species (i.e. *via* the assessment of a species’ genomic repertoire) or entire communities *via* amplicon sequencing^8^. For instance, Gordon and Van Norman^9^ investigated the presence of the rare polypore *Bridgeoporus nobilissimus* (W. B. Cooke) T. J. Volk, Burds. & Ammirati in trees of the Pacific Northwest region of the United States and found evidence for significantly higher abundances of the fungus using DNA sequencing than what was indicated by the presence of fruit bodies in tree species previously unknown to host the fungus. However, no fruit bodies were found on the three additional tree species that tested positively for mycelium, which could be an indication that those occurrences do not necessarily contribute to fruiting and reproduction of the fungus. Similarly, *Phellinus nigrolimitatus* (Romell) Bourdot & Galzin has been found to be present as mycelium in deadwood years prior to forming fruiting bodies^6^. Fungal monitoring could therefore profit from incorporating molecular methods into surveys, as amplicon sequencing could aid in detecting rare species with low fruiting frequency^10^. However, the reliability of amplicon sequencing as a detection method for species from environmental DNA remains the subject of debate, as errors and biases may be introduced during the laboratory, sequencing or bioinformatics workflows, leading to false-positive identification or overlooking of species due to amplification or sequencing errors or incomplete databases^11,12^. Additionally, amplicon sequencing is not able to deliver reliable information on species abundances^8^. One possibility to alleviate these pit falls is combining amplicon sequencing with targeted quantitative (q)PCR for either an entire phylum or selected species using highly specific primers to gather more reliable information on abundances and/or biomass^6^.

Based on fruit body surveys, *Phellinidium pouzarii* (Kotl.) Fiasson & Niemelä is classified as an exceptionally rare wood-inhabiting fungus from the order *Hymenochaetales* known from only seven locations worldwide (Austria, Czechia, Croatia, Germany, the Russian Caucasus area, Slovakia and Ukraine^13–15^). Due to its rarity and the restriction of its occurrence to old-growth forests, *P. pouzarii* is listed on the national Red Lists of Austria^16^, the Czech Republic^17^, Germany^18^ and Slovakia^19^ and has been suggested as an indicator species for highly preserved old-growth forests^13^. In Germany, its occurrence remains restricted to the old-growth forest reserve known as Mittelsteighütte in the Bavarian Forest National Park. So far, no fruit bodies have been recorded outside this reserve in Germany. *P. pouzarii* has been reported to almost exclusively grow on tree trunks of decomposing firs (*Abies alba* (Mill.) and *A. nordmanniana* (Stev.) Spach) in intermediate decay stages and altitudes between 710 and 1100 m above sea level (a.s.l.)^13^ The species has also been found on recently dead *Abies* logs, raising the question of whether it might have been present as an endophyte or parasite prior to the tree dying^13^. The scent given off by the resupinate basidiomata is reminiscent of sweet honey, rose, syringa or hyacinth blossoms and has been compared to that of perfumed soap^20^. Possible ecological functions of this smell have so far not been investigated. Within in the Bavarian Forest, efforts have been made to support *P. pouzarii*’s population *via* inoculation of recently fallen fir deadwood with wooden dowels overgrown with the species’ mycelium^21^ - so far with little success (personal information).

Here, our goal was to gain a first insight into *P. pouzarii’*s status in the Bavarian Forest and add to the sparse ecological knowledge of the species in order to inform future conservation strategies. We aimed to answer the following sets of questions in our study:

1. What is the current population status of *Phellinidium pouzarii* in the Bavarian Forest, and what are its possible ecological limitations?
2. What is the specific lifestyle of *P. pouzarii*, and what are the main compounds of its specific odour?

To address our first question, we conducted a fruit body survey in the Bavarian Forest National Park and sampled *Abies alba* deadwood logs within three regions of the park. Based on previous works^6,9^, we expected to detect *P. pouzarii* more frequently using a molecular approach than what could be derived by fruit body surveys. Based on literature, we concentrated our efforts on *A. alba* logs in intermediate stages of decomposition as assessed in the field. In addition, we assessed the co-occurring fungal and bacterial communities to clarify whether samples containing *P. pouzarii* differed significantly in terms of microbial community composition.

To answer our second set of questions, we cultivated *P. pouzarii* in the laboratory, sequenced its genome using shotgun sequencing, analysed the chemical substances mainly responsible for its characteristic scent, and searched for genes encoding enzymes relevant for their synthesis as well as genes relevant for wood degradation.

## Results

### Genome sequencing and enzymatic repertoire

In total, 4.7 million raw reads were generated during sequencing, which were quality trimmed to 4.13 million reads. The *de novo* assembled genome contained 1,776 contigs with a total size of 28.6 mega base pairs (Mbp) and a G + C content of 47.1%. Estimated coverage amounted to 28×, the largest contig included 261,763 bp. Assembly quality as assessed by *N*_50_ and *L*_50_ values yielded 41,383 and 204, respectively. Single-copy ortholog analysis reported a genome completeness of 91.6% (complete BUSCOs).

Gene prediction identified 8,701 protein-coding genes. 503 Carbohydrate-active enzymes (CAZymes) and related binding modules were found in the genome of *P. pouzarii* using dbCAN3, among them 153 glycoside hydrolases (GHs), 18 carbohydrate esterases (CEs), 8 polysaccharide lyases (PLs), 42 glycosyltransferases (GTs), 63 enzymes with auxiliary activities (AAs, i.e. various oxidoreductases), and 39 carbohydrate-binding modules (CBMs), 11 of which belong to CBM1 that binds to cellulose (Supplementary Figure S3.1). Of these CAZymes, 128 belong to the group of plant cell-wall degrading enzymes (PCWDE^22^) acting on lignocellulose, including 64 GHs, 7 CEs, 52 AAs and 12 versatile lipases (VLPs).

Manganese peroxidases (MnPs) represent true ligninolytic class II peroxidases (PODs), whose presence is indicative for a white-rot lifestyle. We unambiguously identified nine putative MnPs within the genome of *P. pouzarii*, of which eight were classified as so-called long MnPs (containing an additional disulphide bridge at the C terminus) and one as a short MnP^23^ (Supplementary Figure S3.9). All except one (gene g6667) showed the three typical acidic amino acids (EED motif) forming the binding pocket for Mn^2+^ ions (to be oxidized). In no case was a catalytic tryptophan found, which occurs in lignin and versatile peroxidases.

A copy number analysis of the ITS region revealed an approximate copy number of 75 (mean coverage ITS = 1,882×, median coverage single copy gene containing contig = 25×).

**Fig. 1:**
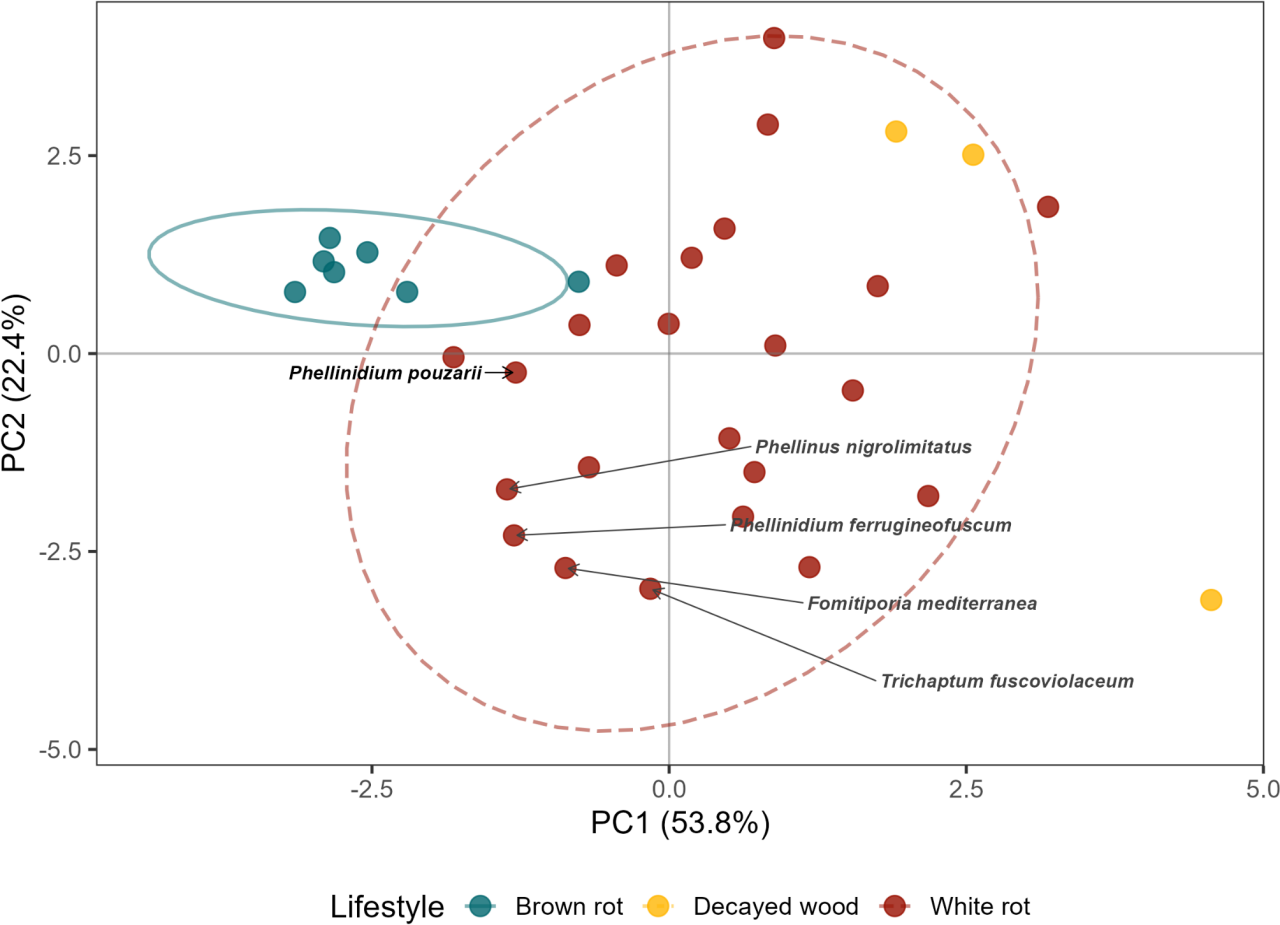
Principal Components Analysis (PCA) of fungal plant cell-wall degrading enzymes (PCWDE), with the position of *P. pouzarii* highlighted; species belonging to the order *Hymenochaetales* are also labelled with their scientific name. Colours and ellipses indicate fungal lifestyle: blue, solid line – brown rot, yellow – decayed wood (not enough points for ellipse), red, dashed line – white rot. First two axes of the PCA are displayed.

To compare *P. pouzarii’*s enzymatic repertoire to that of other wood-degrading fungi from temperate forests, we conducted several PCAs based on two additional data sets^22,24^ separated by the polymeric substrates cellulose, hemicelluloses and lignin (Fig. 1, Supplementary Figure S3.12). All PCAs showed a clear separation of brown-rot and white-rot fungi with *P. pouzarii* clustering within the group of white-rot fungi. Within the PCA based on cellulose-degrading enzymes only, CBM1 (7.28) and AA9, a lytic polysaccharide monooxygenase (LPMO, 3.39), had the highest positive loadings within the PC1 axis. For the hemicellulose data set, the highest loadings in PC1 occurred for VLPs (4.09) and CE16 (0.84). In the PCA performed on lignin-modifying enzymes, class II peroxidases (AA2, 0.58) and H_2_O_2_-forming glucose-methanol-choline oxidase/dehydrogenases (AA3, -5.84) had the highest positive and negative loadings, respectively.

### Fragrance

**Fig. 2:**
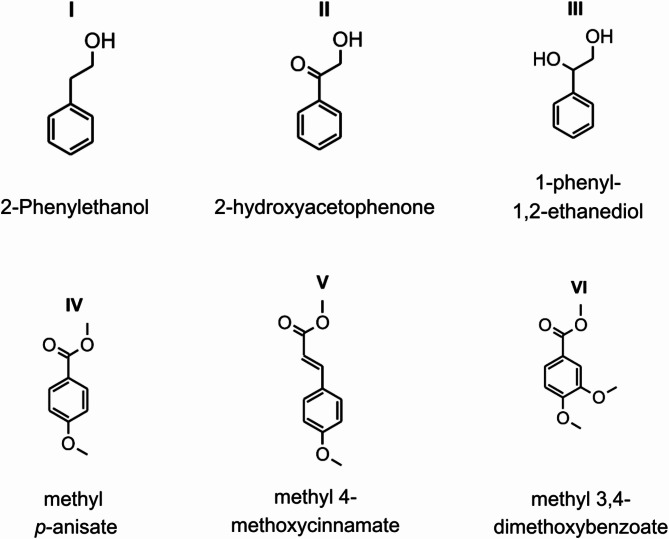
Structural formulae of aromatic compounds detected in organic solvent extracts from a fruit body and fir-wood colonized by *P. pouzarii*.

GC-MS and LC-MS-based analyses of organic extracts of a fruiting body of *P*. *pouzarii* and *A. alba* wood colonized by the fungus revealed the presence of various aromatic compounds (Fig. 2, Supplementary Figure S3.8a-c). Compounds I to VI were detected in the fruit body (VI only by GC-MS; Table 1). The compound with the highest concentration was 2-hydroxyacetophenone (II) with 16.7 mg g^− 1^, which is an aromatic compound also found within plant tissue with antibacterial properties^25^, followed by 1-phenyl-1,2-ethanediol (III) with 7.73 mg g^− 1^. 2-phenylethanol (I), methyl *p*-anisate (IV) and methyl 4-methoxycinnamate (V) were detected in lower but still considerable concentrations of 2.0 to 2.5 mg g^− 1^, while methyl 3,4-dimethoxybenzoate (VI) was only detectable in traces by GC-MS. Analysis of the fir-wood sampled near the fruiting body resulted in the identification of compounds I to IV, whereby IV was merely present in traces and could not be quantified. Compounds I, II and IV were detected in an amount of about 1 mg g^− 1^, which is lower than in the corresponding fruiting body. The substance most perceptible to humans in the extracts (and already in the original samples) was 2-phenylethanol, which was confirmed by olfactory testing of the corresponding authentic standards. We found several genes connected with synthesis of the main components within the *P. pouzarii* genome (Supplementary Table S1.4). For an in-depth discussion of possible synthesis pathways of components, please compare supplement S1.

**Table 1:** Amounts (mg g^− 1^ dry mass) of relevant aromatic compounds from a fruit body and surrounding wood colonized by *P. pouzarii*.

Sample	I	II	III	IV	V
Wood	0.94 (± 0.02)	1.17 (± 0.01)	*	1.41 (± 0.05)	*
Fruit body	2.43 (± 0.43)	16.67 (± 1.66)	7.73 (± 0.99)	2.03 (± 0.36)	5.65 (± 0.81)

Substances were extracted with acetone and quantified by HPLC-DAD (mean values ± standard deviation); N.d.: not detectable; *: below limit of quantification.

### Molecular community composition

**Fig. 3:**
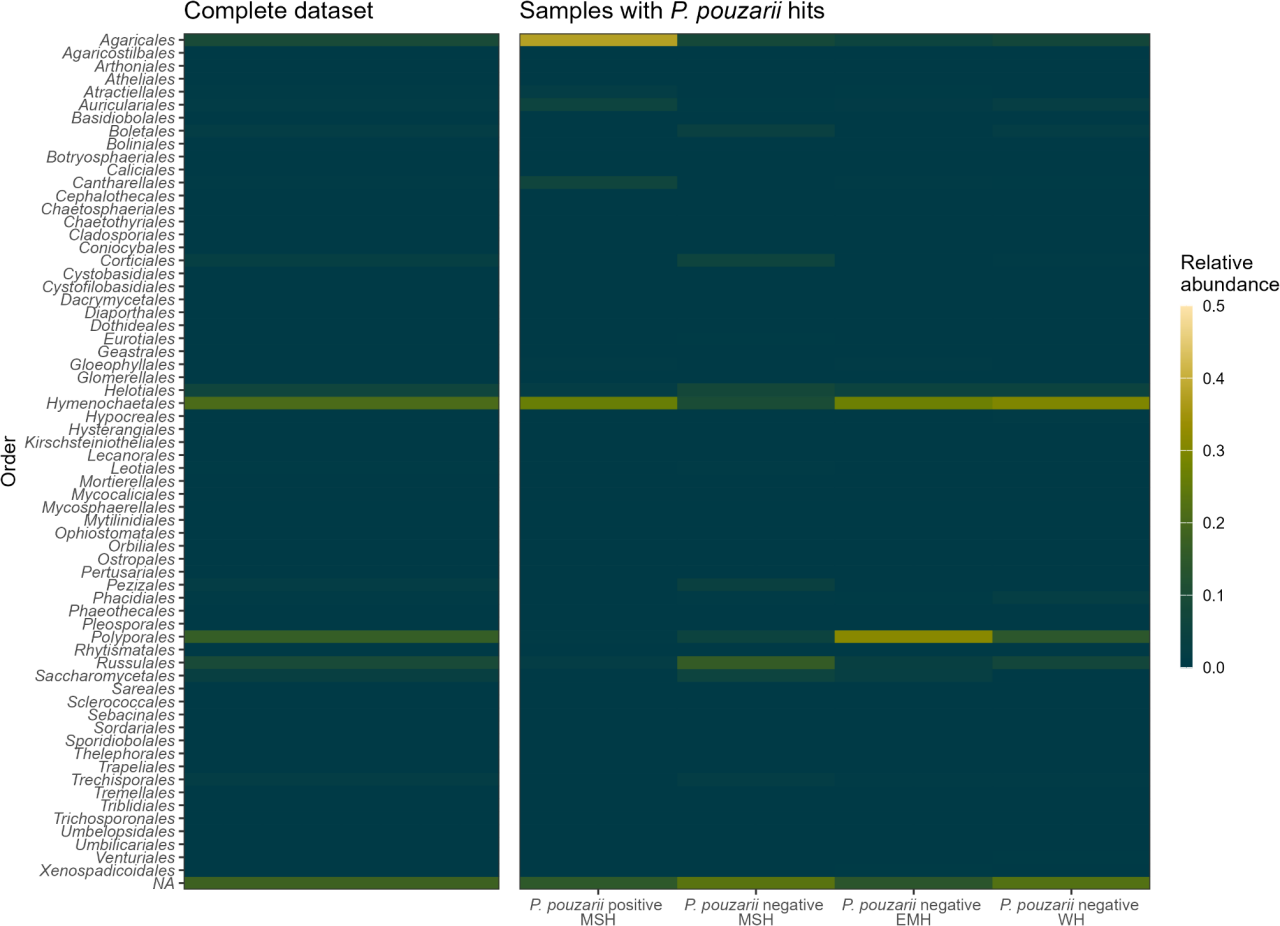
Fungal molecular community composition on the order level across the entire dataset (left) and split across regions and the detection of *P. pouzarii* (right).

943 fungal OTUs (1,969 ASVs pre-clustering) were generated from 9.9 million raw reads. Seven samples were not considered for the ITS data set as they did not contain fungal PCR products. The three most abundant fungal OTUs in the entire data set belonged to *Hyphodontia pallidula* (relative abundance 6%), *Fomitopsis pinicola* (6%) and *Hyphodontia aspera* (5%, Fig. 3, Supplementary Table S4.2).

**Fig. 4:**
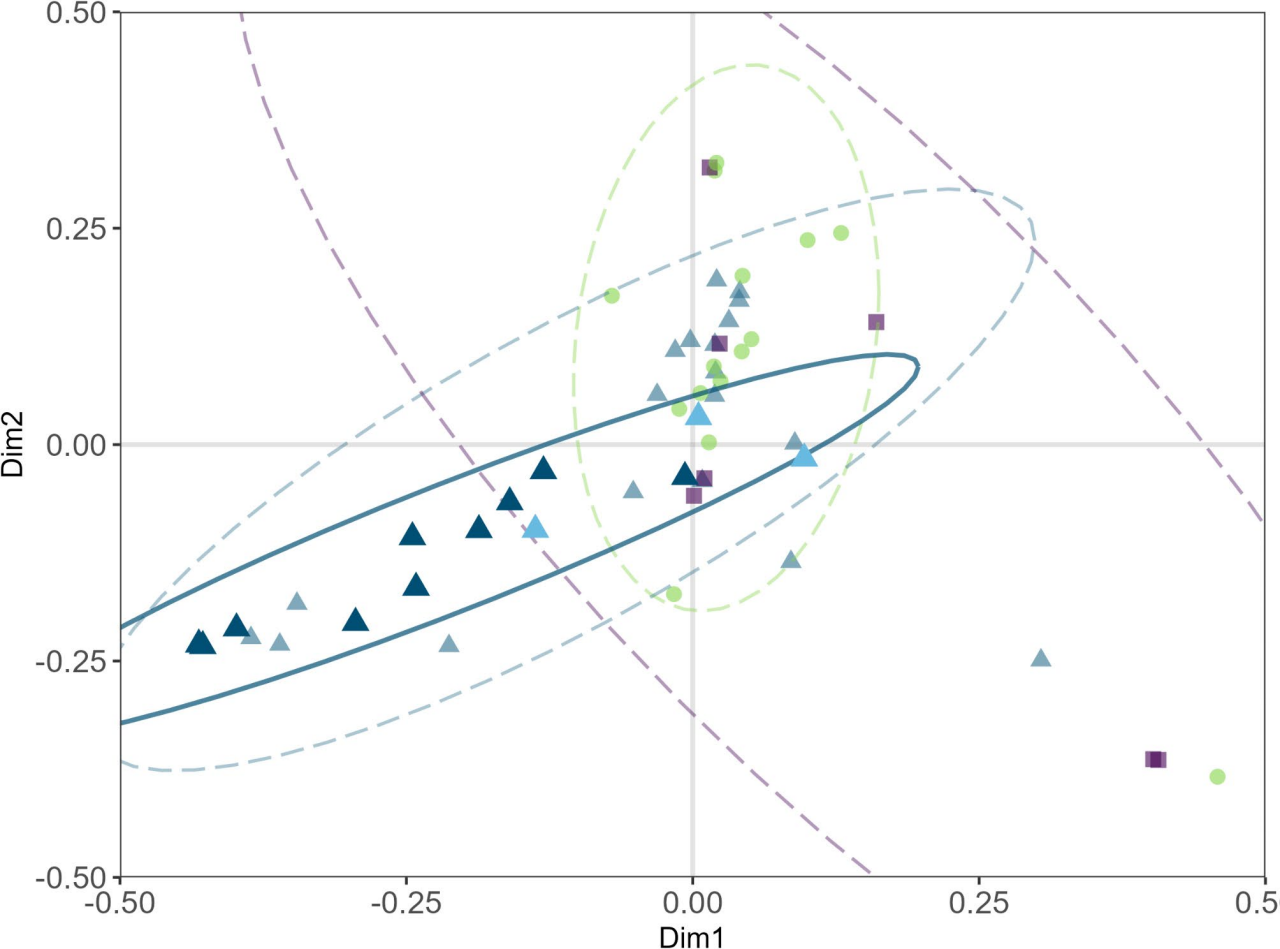
PCoA of fungal communities from 2022 and 2021 including three logs that were sampled several times (TA1, TA12, TA19). Transparent symbols represent samples that tested negatively using amplicon sequencing for *P. pouzarii*: Green circles - Eisenmannhaus (EMH); blue triangles - Mittelsteighütte (MSH); purple rectangles - Watzlik-Hain (WH). Samples that tested positively for *P. pouzarii* using amplicon sequencing are highlighted: Dark blue solid triangles represent 2021 samples TA1, TA12, TA19, light blue solid triangles represent 2022 samples (TA1, TA12).

According to a PERMANOVA, the factor region and the occurrence of *P. pouzarii* had marginally significant effects (p_region_ = 0.09, R^2^ = 0.05; p_*P. pouzarii*_ = 0.06, R^2^ = 0.03; Supplementary Table S4.4) on the fungal community composition in 2022. This translated well to our PCoA including both data sets (2021 and 2022), in which samples clustered according to their sampling region (Fig. 4, Supplementary Figure S3.13). The region Eisenmannhaus had differed significantly in species richness from both Watzlik-Hain (*p* = 0.05) and Mittelsteighütte (*p* = 0.01). This did not occur in a comparison of Shannon indices, where only Eisenmannhaus and Watzlik-Hain differed significantly (*p* = 0.03; Supplementary Figure S3.5).

9.9 million raw reads resulted in 6,267 bacterial OTUs (15,721 ASVs pre-clustering); most abundant bacterial OTUs included *Methylovirgula* (3%), *Conexibacter* (3%) and *Burkholderia* (3%, Supplementary Table S4.3). Region, decay stage and presence of *P. pouzarii* had no effects on bacterial community composition (Supplementary Table S4.4, Supplementary Figure S3.7). Additionally, we found significant differences in bacterial species richness between Eisenmannhaus and Watzlik-Hain (*p* < 0.01), however, this did not translate to Shannon indices (Supplementary Figure S3.5). We found no differences in abundances of gram negative or positive bacterial OTUs in samples containing *P. pouzarii* (p_gram negative_ = 0.81, p_gram positive_ = 0.71; Supplementary Figure S3.10).

### Quantitative PCR

**Fig. 5:**
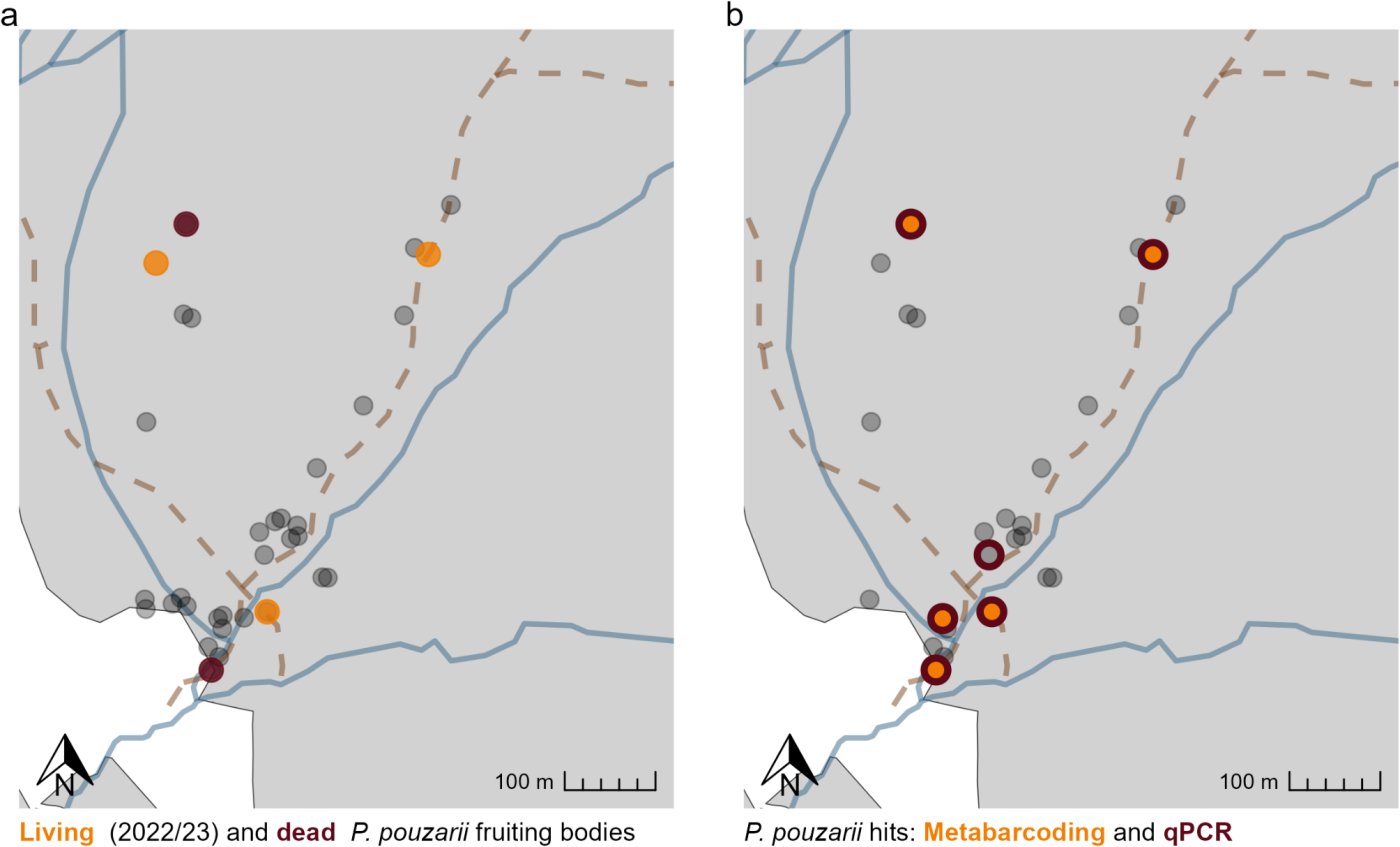
Results of the fruiting body survey (**a**) and the molecular survey (**b**, based on samples from 2021 and 2022) north of Zwiesler Waldhaus (Mittelsteighütte) to detect *Phellinidium pouzarii* in deadwood objects in the Bavarian Forest National Park. Black transparent points indicate logs that were surveyed and sampled, but did not yield positive qPCR or amplicon sequencing results. A: yellow points indicate living (recent) *P. pouzarii* fruit bodies, red points mark *P. pouzarii* fruit bodies that were dead in 2022. B: yellow points mark samples where we detected *P. pouzarii* using amplicon sequencing, red circles indicate that we detected the species using qPCR. Some samples did not contain any fungal DNA and were therefore not included (B). Pathways (brown dashed lines) and streams (blue lines) are displayed for better orientation. Maps of Watzlik-Hain and Eisenmannhaus are not included since we did not detect *P. pouzarii* in those samples.

Of all 81 samples (2021 and 2022) that were initially used for qPCR, 15 yielded positive results. Overall, qPCR and amplicon sequencing results corresponded well with each other, except for one deadwood object, where only the qPCR gave a positive result regarding the presence of *P. pouzarii* (TA29, Fig. 5; Table 2). Our in-depth analysis of three logs with documented fruiting bodies showed a clear pattern of decreasing *P. pouzarii* gDNA with increasing distance from the fruit bodies (Supplementary Figure S3.6). The highest amount of *P. pouzarii* gDNA was found close to a fruiting body with 101.2 ng g^− 1^ dry matter (TA1, 2021 sample, Supplementary Figure S3.6).

**Table 2:** Overview of the sampled Deadwood objects on/in which *P. pouzarii* was detected either as fruit body or by molecular methods (amplicon sequencing, qPCR), including relative abundance and/or the calculated concentration of genomic DNA (gDNA) per gram dry mass (DM).

Object no.	Year	Fruit body status	Relative abundance (%)	gDNA concentration (ng g^− 1^ DM)
TA1	2022	Dead	0.0	0.2
TA1_1^a^	2021	Alive	21.1	101.2
TA1_2^a^			1.3	2.4
TA1_3^a^			19.1	7.2
TA1_4^a^			-	0.0
TA1_5^a^			0.0	0.0
TA6	2022	None	1.7	5.9
TA12	2022	Alive	0.1	0.4
TA12_1^a^	2021	Alive	0.0	20.2
TA12_2^a^			7.5	7.6
TA12_3^a^			10.8	0.0
TA19	2022	Alive	3.7	15.6
TA19_1^a^	2021	Dead^b^	15.8	25.4
TA19_2^a^		Dead^b^	31.0	83.7
TA19_3^a^		Dead^b^	23.6	40.8
TA29	2022	None	0.0	0.6
TA48^c^	2021	None	0.1	5.5

^a^ Subsamples were taken in increasing distance from the *P. pouzarii* fruit body.

^b^ presumably thought as dead in 2021, but after an unknown incident, several fruiting bodies were newly identified in 2022.

^c^ tested negatively for *P. pouzarii* in 2022.

50 logs tested negative for *P. pouzarii* using amplicon sequencing (seven samples did not contain enough fungal DNA).

### Distribution of *Phellinidium pouzarii* in the Bavarian forest

Using amplicon sequencing, we detected sequences that were identified as *P. pouzarii* in four samples from four logs in 2021 and three logs from 2022, including samples from logs where no fruit bodies had previously been recorded (Fig. 5). *P. pouzarii* was identified in two sample sites in 2021 (TA1 and TA48), which gave negative results in 2022, while a new object sampled only in 2022, yielded positive results (TA6). Our qPCR approach yielded positive results for four deadwood objects in 2021 and six objects in 2022 (Fig. 5; Table 2), which matched the amplicon sequencing results except for one sample (TA4). All samples in which we were able to detect *P. pouzarii* by amplicon sequencing or qPCR originated from the Mittelsteighütte region; we found no evidence of *P. pouzarii* in samples from the other two regions.

Besides our target species, we identified several other rare fungal species included in the Red Lists (category R, 1, 2) of Germany, Czechia and/or Austria using amplicon sequencing (Supplementary Figures S3.3 and S3.4).

## Discussion

To answer our first question, i.e. what is the current population status of *Phellinidium pouzarii* in the Bavarian Forest, and what are its possible ecological limitations, we tried to map the present distribution of *P. pouzarii* in the Bavarian Forest using amplicon sequencing and qPCR as well as results from a fruit body survey. Based on our results, *P. pouzarii* seems currently to be restricted to the old-growth forest of Mittelsteighütte. Our molecular approaches gave consistent results, and in most cases, we detected strong molecular signals in logs where fruit bodies had previously been recorded. Contrary to our expectations, we found no evidence of hidden mycelial reservoirs of *P. pouzarii* in or on logs without fruit bodies, except for one log. Our results are therefore an alarm signal regarding the population status of this fungus in the Bavarian Forest and beyond, since only seven populations have been recorded world-wide.

The in-situ conditions under which fungal species (rare and common ones, except those that are commercially cultivated) form fruit bodies are still nebulous^26^. For the specialized *Hymenochaetales* species *Phellinus nigrolimitatus*, a substantial time lag between detection of the fungus by amplicon sequencing and the emergence of visible fruit bodies was reported^6^, i.e., DNA abundance (as a proxy for biomass) and fructification probability did not correlate. For *P. pouzarii* it has been hypothesized that the species might be present in living *A. alba* trees since fruit bodies have been found on recently dead trees. Because *P. pouzarii* fruit bodies have so far only been reported from deadwood, we did not sample any living *A. alba* trees, however, we did include logs from earlier decay stages into our survey and found no evidence for *P. pouzarii* mycelium in those logs. This might be due to the species’ already very few occurrences within our study region and the fraction of *Abies* logs therein we were able to sample. Possibly similar studies using amplicon sequencing and/or qPCR in e.g. the Boubín^13^ or Žofín^13,27^ reserves in the Czech Republic, where the fungus is more abundant, would help to solve this question in the future.

A combination of amplicon sequencing and fruit body inventories for other wood-inhabiting or soil fungi has previously been applied^6,27–29^. Comparisons of amplicon sequencing against fruit body inventories tend to show that many fungal species are present as mycelium within a habitat years prior to forming fruiting bodies^30^. Moreover, molecular inventories do not require the expert taxonomic knowledge necessary for fruit body surveys^27^. However, amplicon sequencing workflows contain various steps that may introduce biases and errors leading to false positive detection of species, such as amplification errors during PCR and sequencing, incomplete or wrong database entries and differences in bioinformatic procedures^11,12^. Here, we validated our amplicon sequencing results by additionally performing targeted qPCRs. Still, molecular detection of target species is not capable of distinguishing between DNA from alive or dead organisms, therefore, we interpret our results with reservation. Nevertheless, the high amount of overlap between fruit bodies and molecular survey points towards reliable results. Therefore, we think that species-specific qPCR surveys could be valuable tools in the conservation of rare wood-inhabiting fungi, as they are comparatively easy to perform and cost-effective.

Nordén et al.^31^ investigated the appearance of generalist and specialist fungal species in fragmented forests in Finland and Russia. The authors reported that overall, rare species struggled in highly fragmented forests, which was attributed to high resource usage specialization (e.g., deadwood of certain tree species, decay classes and minimum diameters of logs/branches). To our best knowledge, there is currently no strict classification of *P. pouzarii* as either specialist or generalist, although it seems to occupy a very specific niche (strong *Abies* spp. deadwood in old-growth forest reserves). While it has been hypothesized that *P. pouzarii* should be expected within its entire possible geographic range, Holec et al. (2019) classify this claim as overly optimistic^13^. They argue that based on fruit body surveys, this species appears to be restricted to old growth and/or virgin forests, of which only a few remain in Europe^32^, rendering it effectively a specialist species, although its genomic properties suggest that it could well be able to decompose other tree species than *Abies*.

Holec et al. (2019) report that *P. pouzarii* seems to favour a microhabitat of naked and/or fractured fir deadwood^13^. This corresponds to our coincidental find of several previously hidden fruit bodies on fractured wood in 2023 (Supplementary Figure S3.2).

Translocation *via* inoculation might be simple but effective tools to support populations of declining saprotroph fungal species^33^ and *P. pouzarii* has already been chosen as a target species for such efforts, although similar attempts have so far not been successful. Nevertheless, the Bavarian Forest offers several possible habitats. The forest area Watzlik-Hain for example seems to be a more or less optimal area for the fungus with its high amounts of strong *A. alba* deadwood logs and less than 2 km distance from Mittelsteighütte, where all currently known *P. pouzarii* fruit bodies in the Bavarian Forest are located. However, we found neither fruit bodies nor molecular evidence for the species in nine suitable samples, which raises fundamental questions about the dispersal mechanism of *P. pouzarii* and its limitations. Comparatively, another rare polypore species, *Antrodiella citrinella* Niemelä & Ryvarden, was shown to easily bridge distances of up to 30 km and establish successfully on Norway Spruce (*Picea abies*), following a large-scale forest dieback caused by *Ips typographus* in the Bavarian Forest National Park^34^.

This leads to our second research question, i.e. what is the specific lifestyle of *P. pouzarii*, and what are the main compounds of its specific odour:

We identified the substances mainly responsible for the fungus’ odour as 2-phenylethanol and methyl *p*-anisate. 2-phenylethanol occurs naturally in a variety of organisms and is a major component of several essential plant oils, including rose and hyacinth flowers^35^. It is also partly responsible for the pleasant odour of young fruiting bodies of *Laetiporus (Polyporus) sulphureus* (Bull: Fr.) Murrill^36^. Methyl *p*-anisate, the second odour-forming substance of the scent of *P. pouzarii*, was described as a component of the distinct anise-like smell of *Trametes suaveolens* (L.: Fr.) Fr.^37^ and *Hydnellum suaveolens* (Scop.: Fr.) P. Karst^38^, and resembles the aroma of *Feijoa sellowiana* (O. Berg) Burret fruits (pineapple guava) that contain methyl and ethyl benzoates^39^. The synthesis of such benzene derivatives by fungi has not yet been linked to interactions with other organisms^40^.

We propose two possible ecological advantages *P. pouzarii* could achieve through the synthesis and secretion of 2-phenylethanol based on available literature. First, the strong rose-like scent may act as an insect attractant, helping to spread the clamydospores and/or the basidiospores. For example, volatile organic compounds produced by *Aureobasidium pullulans* (De Bary) G. Arnaud, including 2-phenylethanol, were shown to attract several insect taxa in an agricultural landscape^41^, where traps baited with 2-phenylethanol resulted in one of the highest diversity scores of captured insects in the entire study. In connection with the fact that we did not find *P. pouzarii* in the Watzlik-Hain forest within 2 km distance from Mittelsteighütte, it would be valuable to further investigate this hypothesis e.g. using insect traps baited with 2-phenylethanol. Alternatively, examining *P. pouzarii*’s dispersal mechanism using spore traps would be equally interesting in order to gain more insight into its possible limitations within the national park. Second, fungal volatile organic compounds may be part of a “chemical strategy” of saprotrophic fungi (in the sense of fungal allopathy) to gain superiority over competitors in resource utilization, e.g., by producing secondary metabolites with antimicrobial properties^42^. 2-phenylethanol was demonstrated to inhibit the growth of gram-negative bacteria^43^ and act as a fungicide against agricultural plant pathogens such as *Aspergillus carbonarius* (Bain. Thom)^44^ and *Botrytis cinerea* (Pers.)^45^. We did not find evidence for a similar effect in our deadwood samples, nevertheless, we think that laboratory experiments to support or falsify this hypothesis would be interesting.

In this study, we sequenced and analysed the genome of *P. pouzarii* to gain insight into its genetic repertoire and fungal lifestyle, and to compare the data with those of other wood-rot fungi from temperate forests. With 52 oxidoreductases relevant for lignin modification or degradation, *P. pouzarii* exhibits the common features of white-rot fungi. Within the order of *Hymenochaetales*, which we included as basis of comparison in our analysis, *P. pouzarii* had the highest number of plant-cell wall degrading enzymes (PCWDE, 71), which was much higher than those for the two closest related species *Fomitiporia mediterranea* (M. Fisch) and *Phellinidium ferrugineofuscum* (P. Karst) Bourdot & Galzin (both with 36 genes), but still significantly lower than in strong white-rot fungi such as *Armillaria mellea* (Vahl) P. Kumm (295 genes). Noteworthy, *P. pouzarii* had the lowest number of cellulose-binding modules (11 CBM1) within the order *Hymenochaetales*.

Based on our analysis, we can say that *P. pouzarii* has a typical white-rot enzymatic repertoire, which would theoretically enable it to degrade several different types of wood across decay stages. However, compared to other white-rot fungi known to cause strong and fast rotting, it has a low number of PCWDE and CAZymes, low number of genes and low copy number of the ITS, i.e. rRNA gene region as well as a slow growth in the laboratory. Correlations between copy number of the ribosomal cistron and fitness have been demonstrated for bacteria, but are currently not well-supported for fungi^46,47^. Additional long read sequencing and hybrid assemblies could improve the current draft genome and facilitate future omics studies.

## Conclusion

Here, we presented an in-depth investigation of *P. pouzarii*’s population status in the Bavarian Forest, its genomic properties and possible ecological strategies. We show that its conservation status in the national park is dire, since we did not find any indication of hidden reservoirs of *P. pouzarii*’s mycelium. Overall, combining qPCR and amplicon sequencing produced overlapping results and we argue that qPCR using species-specific primers could be a valuable tool for the conservation of rare fungi. The results of our genomic analysis show that *P*. *pouzarii* has the typical enzymatic repertoire of a white-rot fungus that should in theory make the species a well-equipped wood-rotter. We characterized the main components responsible for *P. pouzarii*’s distinct smell as 2-phenylethanol and methyl-p-anisate. Further research is needed to investigate if whether the production of these aromatic substances play any role in e.g. dispersal or competition of the fungus.

## Materials and methods

### Study site

**Fig. 6:**
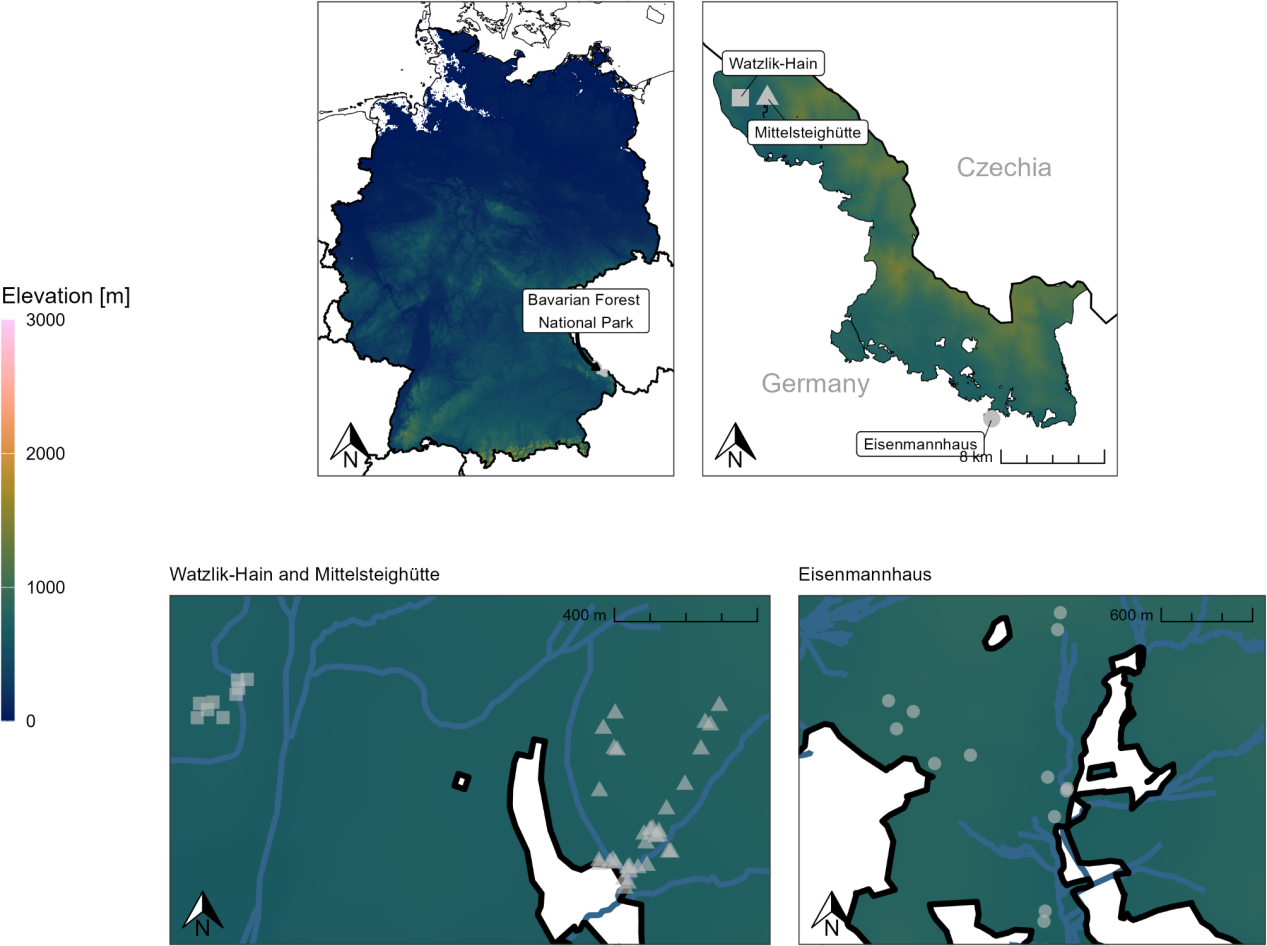
Map of the location of the Bavarian Forest National Park in Germany (top left) and the sampled areas within the national park (top right). Bottom: close-up maps of sampling locations, grey dots represent sampled deadwood objects: Watzlik-Hain (rectangles, 9 deadwood objects) and Mittelsteighütte (triangles, 33 objects) bottom left; bottom right: Eisenmannhaus (circles, 19 objects). Colour displays elevation in m above sea level; water bodies (blue lines) were added for orientation.

The Bavarian Forest National Park is located in south-eastern Germany between 650 and 1450 m a.s.l. next to the Czech border (Fig. 6), on the other side of which lies the Czech Bohemian Forest National Park, forming one of the largest coherent forest areas in Central Europe^48^. Most stands in the park are dominated by Norway spruce (*Picea abies* (L.) H. Karst) stands or mixed mountain forests consisting of European silver fir (*Abies alba* Mill.), Norway spruce and European beech (*Fagus sylvatica* L.)^49^. The national park hosts several remote old-growth forests, such as the Mittelsteighütte close to the village Zwiesler Waldhaus. Since *P. pouzarii* fruit bodies are almost exclusively known to grow on silver fir deadwood in medium or late stages of decomposition^13^, we additionally sampled in forest stands close to the National Park Centre “Eisenmannhaus” and in the Watzlik-Hain close to Mittelsteighütte, where high amounts of silver fir deadwood occur (Fig. 6).

Living fruit bodies of *P. pouzarii* have previously been documented at five logs and verified by sequencing of the internal transcribed spacer (ITS) region, and served as starting material for isolation of mycelial pure cultures. Interestingly, in 2023, a disturbance of unknown cause occurred on one of the logs (TA19), which previously appeared to harbour a fruiting body that was about to die (Supplementary Figure S3.2). However, due to the disturbance (complete position change of the log), several other viable *P. pouzarii* fruit bodies were found on the side of the log, which also exhibited the characteristic perfume-like scent.

### Sample collection

In August 2021, we sampled wood of eight deadwood objects where living or dead *P. pouzarii* fruit bodies had been recorded previously. For context, dead fruit bodies of *P. pouzarii* tend to lose their smell, become darker and drier and, in some cases, fall off their respective substrate. In May 2022, we resampled five of these logs (TA1, TA12, TA19, TA23, TA48) as well as 52 additional randomly selected lying *Abies alba* deadwood objects across four decay stages on three sites in the Bavarian Forest National Park (Fig. 6). Decay stages were assigned in the field according to Heilmann-Clausen (2001): Decay stage 1 for recently dead logs with intact and hard wood and twigs; 2 for still hard wood, but bark that is falling off; 3 for softer wood, nearly all bark gone, but intact log diameter; 4 for strongly decayed and spongy deadwood with a reduction in diameter and decay class 5 for almost completely decayed wood, original shape of the log is lost^50^. Deadwood samples were taken by drilling into the logs using a wood auger (Makita, Anjō, Japan; d = 1 cm, l = 40 cm), which was flamed and wiped down with alcohol in between drillings to prevent contaminations. In order to determine the DNA content in the wood in vicinity to a fruit body, logs TA1, TA12 and TA19 (all located at Mittelsteighütte), on which fruit bodies had already been recorded, were sampled repeatedly 2021, first at a distance of approx. 10 cm from the respective fruit body as the closest sample point and then successively at a distance of 1 m (up to 4 m away, Supplementary Figure S3.11). Samples were collected in sterile bags, transported to the lab at -20 °C and stored at -80 °C until further processing. Portions of samples were pre-processed by milling with the addition of dry ice (analytical mixer mill MM400, Retsch, Idar-Oberstein, Germany).

### Fragrance isolation and Elucidation

We extracted the volatile organic substances from a fresh wood sample and a fruit body of *P. pouzarii* from the same log (TA19); both were not dried and kept at -18 °C prior to extraction. Corresponding samples were dried to determine the moisture content and to calculate the dry matter content. Both sample types were cut into small pieces (< 3 mm), weighed (1.7 g wood sample, 0.3 g fruit body sample) and transferred into reaction tubes. The extraction solvent dichloromethane (VWR Chemicals, Delaware, USA) for gas chromatography-mass spectrometry (GC-MS) or acetone (Merck, Darmstadt, Germany) for high performance liquid chromatography-mass spectrometry (LC-MS)) was added at the ratio of 1:3.25 and 1:6.5, respectively. Samples were mechanically stirred for 6 h and then subjected to an ultrasonic treatment for 30 min (Sonorex, Bandelin, Berlin, Germany). Subsequently, reaction tubes were centrifuged (8,000 rpm, 20 min) and liquid extracts removed. For details and a full list of authentic standards, please compare supplement S1.

### Genome sequencing and phylogeny of class II peroxidases

A *P. pouzarii* strain (IHI 569, DSM 108285) was isolated from fruit body pieces growing on *Abies alba* deadwood in October 2014 from Mittelsteighütte at the Bavarian Forest National Park. The slow growing isolate was cultured on malt-extract agar plates and biomass was scraped off several plates for genomic DNA (gDNA) extraction. gDNA was extracted following a standard cetyltrimethylammonium bromide (CTAB)-based method^51^ prior to sonographic shearing using a S2 ultrasonicator (Covaris, Woburn, USA). A 200-bp library was constructed using the Ion Plus Fragment Library Kit (ThermoFisher Scientific, Darmstadt, Germany) and the library was sequenced on an Ion Torrent Personal Genome Machine (PGM) using the Ion PGM Sequencing 200 Kit v2 and a 318v2 chip (ThermoFisher Scientific).

Resulting reads were trimmed using BBDuk 38.84^52^ (minimum quality = 13 on both ends, minimum length = 180 bp) and *de novo* assembled using MIRA 4.0^53^ (minimum reads per contig = 100, quality level = accurate). Geneious Prime’s^54^ assembler was used with the highest sensitivity to join the contig ends and filter for duplicate contigs. Assembly quality was assessed using QUAST v4.5^55^ and for single-copy ortholog analysis, we used BUSCO 5.5.0^56^ (fungal data set, agaricomycetes_odb10). Gene prediction was performed using the AUGUSTUS 3.4.0 web server (predictor *Laccaria bicolor*)^57^. Carbohydrate-active enzymes (CAZymes) were identified using dbCAN3 (HMMdb v12; E value, <1e^− 15^; coverage > 0.35)^58^. Specific enzymes, such as unspecific peroxygenase (UPO, EC 1.11.2.1), dye-decolorizing peroxidase (DyP, EC 1.11.1.19) and versatile lipase (*Candida rugosa*-like lipase family abH03.01, VLP) were identified using blastp searches of the predicted proteins with reference sequences^59^. Ligninolytic class II peroxidases, i.e. manganese peroxidase (EC 1.11.1.13) as strongest indicator for white-rot physiology as well as lignin peroxidase (EC 1.11.1.14) and related enzymes were aligned using Clustal Omega 1.2.2^60^, and the phylogeny analysed using a maximum-likelihood approach integrated in RAxML^61^ with 500 bootstrapping replicates (settings: protein model GAMMA BLOSSUM62, algorithm Rapid Bootstrapping and search for best-scoring ML tree).

The rRNA gene operon including 18S rRNA gene, internal transcribed spacer (ITS) 1, 5.8S rRNA gene, internal transcribed spacer 2 and 28S rRNA gene was identified using a blastn search^59^ against all contigs using an *P. pouzarii* reference ITS sequence. The analysed ITS sequence served as the basis for the development of specific primers used in the quantitative PCR approach. To assess the copy number of the ITS region, we followed a remapping approach by remapping all raw reads against all contigs and calculating the coverage. The ITS copy number was calculated by division of the mean coverage of the ITS region and the median coverage of all contigs (i.e. thereby representing a contig with single copy genes and non-repetitive sequences).

### Molecular community analysis of deadwood samples

DNA was isolated from 0.2 g milled wood samples with the Quick-DNA Fecal/Soil Microbe Kit (Zymo Research, Irvine, USA) following the manufacturer’s protocol.

For amplicon sequencing, marker regions ITS2 and 16S were amplified for the identification of fungi and bacteria, respectively, by using the primer combinations fITS7 (forward)/ITS4 (reverse)^62^ and 515F (forward)/806R (reverse)^63^ following published protocols^64,65^ (Supplementary Table S2.1 and S4.1). Target PCRs were performed in duplicates and products were pooled prior to purification (PCR Purification Kit, Analytik Jena GmbH & Co. KG, Jena, Germany) and quantification by UV-spectrophotometry (ND-2000 C, ThermoFisher Scientific). In the preparations for sequencing, an additional index PCR was performed using the Nextera XT Library Preparation Kit (Illumina, San Diego, USA)^66^. Final libraries were sequenced on an Illumina MiSeq platform in the mode 2 × 300 bp paired-end at the Department of Soil Ecology of the Helmholtz Centre for Environmental Research (UFZ), Halle (Saale).

### Quantitative PCR approach

For the qPCR approach, two new specific primer pairs for the ITS region were developed and tested (Supplementary Table S4.1). Using gDNA of *P. pouzarii* and a selection of *Basidiomycota* as negative control, the primer pair PhePou_455F & 634R proved to be suitable for the use in qPCR. We validated the PCR product by purifying and sequencing a ~ 180 bp product, which was 100% identical with the *P. pouzarii* ITS sequence.

To assess the amount of *P. pouzarii* DNA present in the samples, a pure culture of *P. pouzarii* was cultivated and its DNA isolated. To determine the nucleic acid concentration, the sample was analysed with a Qubit dsDNA HS Assay Kit (ThermoFisher Scientific) and a Qubit 2.0 Fluorometer. The DNA stock concentration was 6.4 ng µl^− 1^. For quantification, a standard curve was prepared using 10-fold serial dilutions of the *P. pouzarii* DNA with 1:1000 as lowest, which resulted in the following equation:

$\:y\:=\:{874912e}^{-0.643x}$ (where the x equals the measured C_t_ value).

All samples that yielded gDNA were used for PCR amplification with the specific primers. qPCR reactions were performed in triplicates, whereas all negatives were only measured once since we did not expect any amplification. Quantitative PCR was carried out at a Rotor-Gene Q (Qiagen, Venlo, Netherlands) using the PowerUp™ SYBR™ Green Master Mix (ThermoFisher Scientific) following the manufacturers manual with a total reaction volume of 20 µl (10 µl Sybr Green Master Mix, 7 µl water, 1 µl forward primer (10 pM), 1 µl reverse primer (10 pM) and 1 µl DNA template). In brief, the following PCR program was used: 50 °C for 2 min, initial denaturation at 95 °C for 2 min, 40 cycles of 95 °C for 20 s, 52 °C for 20 s and 72 °C for 1 min with acquiring on green (source: 470 nm, detector: 510 nm). A melting curve (stepwise increase of 1 °C from 72 °C to 95 °C, after a 90 s pre-melt conditioning, holding each step for 5 s) verified the specificity of the PCR product. The amount of *P. pouzarii* gDNA per g dry weight was calculated using fresh weight at extraction and dry weight.

### Amplicon sequencing analysis

Raw read FASTQ files were processed using the Linux-based snakemake implementation dadasnake^67^ (version 0.11.2) of the DADA2 algorithm^68^ (version 1.14). Primer sites and primer sequences were searched and removed from reads using cutadapt^69^ (version 4.1). Denoising, error estimation, chimera removal and merging were all performed using DADA2.

For bacterial amplicons, trunc length was set to 170 bp for forward and 130 bp for reverse reads, with a minimum trunc quality (truncQ) of 13 and a maximum expected error (maxEE) of 0.2. Merging was performed using a minimum overlap of 12 bp with no mismatches allowed. Fungal amplicons were processed with a minimum trunc quality of 15 bp and a maxEE value of 3 to account for the high variability in length and higher error probability in the barcoding region. ITS reads were merged using a 20 bp minimum overlap with two mismatches allowed. Amplicon sequence variants (ASVs) as constructed by DADA2 were then clustered into operational taxonomic units (OTUs) using a 97% similarity cutoff with VSEARCH^70,71^. 16 S sequences with less than a minimum length of 245 bp and more than 275 bp length were omitted to filter out mitochondrial and plastid sequences. Taxonomic assignment was performed using the NCBI nt database and the blastn algorithm for ITS sequences^59^. For 16S sequences, we used the mothur algorithm^72^ and the SILVA database^73,74^ (release 138). For functional annotation of fungal taxa, we used the FungalTraits database^75^.

### Statistics

Statistical analysis was conducted using R (Version 4.2.3, R Core Team) and the RStudio IDE (RStudio 2023.09.0 + 463, Posit Software, Boston, USA).

Before further analysis, fungal and bacterial OTUs were filtered for any remaining plastids or non-fungal/non-bacterial OTUs. 7 samples from the fungal 2022 data set that did not contain any bacterial or fungal sequences were excluded as well. Most analyses were performed using OTU matrices transformed to relative abundances, except for the calculation of alpha diversity measures.

To address our first question and compare the two molecular methods we applied, we created maps visualizing the occurrence of *Phellinidium pouzarii*’s fruit bodies and the results of our sequencing and qPCR approach using the R packages *sf*^76^ as well as *osmdata*^77^ and *osmextract*^78^ for additional openstreetmap data (openstreetmap.org). Shape files for the Bavarian Forest National Park were obtained from the Bavarian State Office for Environment (https://www.lfu.bayern.de/umweltdaten/geodatendienste/pretty_downloaddienst.htm?dld=schutzgebiete.xml). Elevation data for Fig. 6 was downloaded from Terrain Tiles (https://registry.opendata.aws/terrain-tiles/) using the *elevatr*^79^ R package.

To test for any effects of the occurrence of *Phellinidium pouzarii* on the co-occurring fungal and bacterial communities, we calculated alpha diversity measures (species richness and Shannon index) using abundance matrices rarefied to 10,000 reads and tested using Wilcoxon tests. Additionally, we tested for differences in fungal communities between regions, decay stages and logs with and without *P. pouzarii* hits using a permutational analysis of variance (PERMANOVA, function *adonis2*, *vegan* package^80^, 999 permutations). To visualize differences between communities across regions and decay stages, we conducted a Principal Coordinates Analysis (PCoA).

Bacterial OTUs were assigned with gram stain information using the AMR package (function *mo_gramstain*^81^) and tested for any differences between relative abundances of bacteria of different gram stain types between samples with and without *P. pouzarii* hits in either qPCR or amplicon sequencing *via* Kruskal-Wallis tests. Homoscedasticity and normality were checked using Levene’s test and Shapiro-Wilk tests.

To address part of our second question, we compared *P. pouzarii*’s genetic repertoire to that of other common saprotroph fungi using available datasets published by Ruiz-Dueñas et al., (2021)^22^ and Zhao et al. (2023)^24^ by conducting a Principal Components Analysis (PCA, function *rda*, *vegan* package) on the numbers of plant cell-wall degrading enzymes (PCWDE) of species included in the dataset. Genes were classified based on the substrate the corresponding enzyme acts upon (lignin, hemicellulose, cellulose). Where necessary, we supplemented those data sets for certain genes using genomes submitted to MycoCosm^82–84^ and a set of three reference genes to search for unspecific peroxidases (UPO), dye-decolorizing peroxidases (DyP) and versatile lipases (VLP). Lifestyles and decay types were checked using the FungalTraits database^75^.

All tests were performed at α = 0.05. For data manipulation and general visualization, we used the *tidyverse* family of R packages^85^.

## Electronic supplementary material

Below is the link to the electronic supplementary material.

Supplementary Material 1

## Data Availability

The ribosomal gene operon of Phellinidium pouzarii used in this study is deposited at GenBank under accession number MK501618. All raw reads, the assembly and predicted genes are submitted to NCBI under the Bioproject PRJNA521454. Fungal and bacterial OTU tables as well as metadata are available under https://doi.org/10.5281/zenodo.13862210. Raw community sequencing data are submitted to NCBI under Bioproject PRJNA1223473.
