# Supplementary material for: Illuminating ecology and distribution of the rare fungus Phellinidium pouzarii in the Bavarian Forest National Park
Source: Sci Rep. 2025 Mar 12;15:8604. doi: 10.1038/s41598-025-91672-y (PMC11904187; doi:10.1038/s41598-025-91672-y)
Supplement: Supplementary file 1 — Supplementary Material 1 [file 41598_2025_91672_MOESM1_ESM.docx]

Supplement:

Illuminating ecology and distribution of the rare fungus *Phellinidium pouzarii* in the Bavarian Forest National Park

## S1: Extraction of a fruit body from *Phellinidium pouzarii* and adjacent wood colonized by the fungus

### Methodology

We sampled splintered wood from a fir log (*Abies alba*) obviously colonized by *Phellinidium pouzarii*, which was evident by several basidiocarps (fruiting bodies) and a distinct rose-like odour that was perceptible for several meters (TA19; figure S1). We also took about 3 g of a well-developed living fruiting body of the fungus from the same fir log TA19. All samples were frozen at -18 C°. Extractions were carried out with freshly thawed material without drying in order to avoid a loss of volatile substances. Corresponding samples were dried to determine the degree of moisture and to calculate the dry matter content of the aromatic substances. Both sample types were manually cut into small pieces (< 3 mm). About 1.7 g and 0.3 g of the wood and fruit body samples were weighed into appropriate reaction tubes (50 or 5 mL) and the extraction liquid was added in ratios of 1:3.25 or 1:6.5, respectively. Dichloromethane served as the extraction solvent for samples that were subsequently analyzed by gas chromatography-mass spectrometry (GC-MS), while acetone or A. dest. or a mixture of both (50/50 vol/vol) were used for high performance liquid chromatography-mass spectrometry (LC-MS) samples. The solid samples suspended in the solvent were mechanically stirred for at least 6 h and subjected to ultrasonic treatment for 30 min (Sonorex, Bandelin, Berlin, Germany). The reaction tubes were centrifuged (8,000 rpm, 20 min) and the liquid extracts transferred into appropriate vials for further analysis.

### Chemicals

Authentic standards of aromatic compounds and the solvents for liquid extraction are listed below:

- 2-phenylethanol ≥99.0% (GC), Sigma-Aldrich, CAS: 60-12-8;
- 2-hydroxyacetophenone >98.0% (GC), TCI Europe, CAS: 582-24-1;
- 1-phenyl 1,2-ethanediol, 97%, Sigma-Aldrich, CAS: 93-56-1;
- methyl p-anisate; ≥99% (FG), Sigma-Aldrich, CAS: 121-98-2;
- methyl 4-methoxycinnamate, 99%, Santa Cruz Biotechnology, Inc., CAS: 832-01-9;
- methyl 3,4-dimethoxybenzoate >98.0%, TCI Europe, CAS: 2150-38-1;
- acetone for analysis, Merck;
- dichloromethane for HPLC, VWR Chemicals

### Gas chromatography / mass spectrometry (GC/MS)

GC/MS was performed on an Agilent 7890A model gas chromatograph equipped with a Gerstel temperature programmed injector (KAS) and a 5975C mass selective detector using a DB5-MS UI (30 m × 0.25 mm ID × 0.25 μm film thickness, Agilent) capillary column at a helium flow rate of 2.0 mL min^-1^. Injection of 0.5 µL DCM extract was done in the splitless mode. Since 2-hydroxyacetophenone has shown to be prone to thermal dehydrogenation, an injector program was used that started at 120°C, held for 0.5 min, and then increased to 160°C with 5°C s^-1^. The column oven was held at 60°C for 4 min and then ramped at 15°C min^-1^ to 300°C. 70 eV EI mass spectra were recorded in the range from 30 to 400 m/z with nominal mass resolution and a rate of 2 Hz. The analytes were identified and quantified by comparison with authentic standards and computer-aided spectrum search (NIST).

### High performance liquid chromatography with diode-array and mass detection (HPLC/DAD-MS)

The acetone and aqueous extracts were analyzed by HPLC using an Agilent Series 1200 instrument system equipped with a diode-array detector (DAD) and an electrospray ionization mass spectrometer (MS; Agilent Technologies Deutschland GmbH, Böblingen, Germany). Reversed phase chromatography was performed on a Synergy™ Fusion-RP column (150 mm x 2 mm length, 4 µm particle size, 80 Å, Phenomenex, Aschaffenburg, Germany), which was eluted at 0.35 mL min^-1^ and 40 C° with aqueous 0.01% (v/v) formate (adjusted to pH 3.5 with an aqueous ammonia solution and acetonitrile / 80:20 v/v) for 0.3 min, followed by a linear gradient to 90% acetonitrile over 8 min. Mass spectrometric measurements were made in the positive ESI mode in a mass range from 40 to 500 m/z (step size 0.1, drying gas temperature 360°C, capillary voltage 4,000 V). The reaction products were identified relative to authentic standards, based on their retention times, UV-Vis spectra, and characteristic mass ions [M + H]^+^.

### Supplementary remarks on the aromatic compounds produced by *P. pouzarii*

#### Methyl p-methoxycinnamate

The final metabolite of the shikimate pathway is prephenate, which can be further converted into arenate, phenylpyruvate and p-hydroxyphenylpyruvate. The enzymes responsible are glutamate-prephenate aminotransferase (PAT; EC 2.6.1.79), prephenate dehydrogenase (PDH; EC 1.3.1.12) and prephenate dehydratase (PDT; EC 4.2.1.51). Phenylpyruvate and p-hydroxyphenylpyruvate are trans-aminated to the corresponding amino acids by GTP (glutamine-phenylpyruvate transaminase; EC 2.6.1.64). In addition, a specific aromatic aminotransferase (AAT I = Aro8; EC 2.6.1.57) is known from the ascogenic yeasts *Saccharomyces cerevisiae* and *Candida* sp. AATs work with glutamate, phenylalanine, tyrosine and tryptophan as amino-group donors and with phenylpyruvate, p-hydroxyphenylpyruvate, 2-oxoglutarate and pyruvate as amino-group acceptors^[1–3]^.

An alternative way of forming tyrosine proceeds *via* the hydroxylation of phenylalanine by a phenylalanine 4-monooxygenase (EC 1.14.16.1; not shown for Eumycota but the slime mold *Dictyostelium*^[4]^. Subsequently, a phenylalanine ammonia lyase (PAL; EC 4.3.1.24) could catalyze the deamination of phenylalanine to cinnamic acid, which in turn would be hydroxylated to p-coumaric acid (p-HCA; p-hydroxycinnamic acid) by a trans-cinnamic acid 4-monooxygenase (TCM; EC 1.14.14.91). The presence of PAL was demonstrated for the basidiogenic yeast *Rhodotorula glutinis* (F.C. Harrison), among others^[5]^. An alternative possibility for the biosynthesis of p-HCA is based on the deamination of tyrosine *via* a tyrosine ammonia lyase (TAL; EC 4.3.1.23). In the next (crucial) step, the carboxyl group is probably methylated with the help of a carboxylmethyl transferase (CMT; EC 2.1.1.x^[6]^). Fungal CMTs have been described for ascomycotal Nectriaceae, i.e. *Fusarium fujikuroi* (Nirenberg) and *Fusarium graminearum* (Schwabe)^[6,7]^; the product formed is methyl-p-hydroxycinnamate (methyl-p-HCA). Further phenolic methylation would lead to methyl p-methoxycinnamate (V), which was detected in larger quantities (5.6 mg per g) in the fruiting body (basidiocarp) of *P. pouzarii*. Fungal methyl-p-methoxycinnamate were observed as the product of a phenolic O-methyltransferase [enzyme preparation of *Lentinus lepideus* (Fr.) (current name *Neolentinus lepideus* (Fr.) Redhead & Ginns; starting from methyl-p-coumarate / methyl-p-hydroxycinnamate)^[7]^. Methyl-p-methoxycinnamate (V) was also detected as a metabolite in the basidiomycotic fungus *Neolentinus lepideus* [Fr., order Gloeophyllales) Redhead & Ginns (as *Lentinus lepideus* (Fr.)] and in the polypore *Trametes suaveolens* (L.) Fr.^[8]^. An O-methyltransferase that is involved in the biosynthesis of aflatoxin was described for *Aspergillus parasiticus*^[9]^. A summarized overview is shown in Figure S1.1.

#### Phenylethane derivatives

The starting point for the enzymatic synthesis of phenylethane (= ethylbenzene) derivatives is phenyl pyruvate, which originates from the fungal shikimate (prephenate) pathway (see above). There are basically two ways to shorten the side chain, both of which are based on the cleavage of CO_2_ by decarboxylases. The first one may start with the decarboxylation of phenylpyruvate to form 2-phenyl acetaldehyde by a phenylpyruvate decarboxylase (PPD; EC 4.1.1.43). The PPD of *Saccharomyces cerevisiae* is a thiamine pyrophosphate-dependent enzyme Aro10^[10]^. The reactive decarboxylation product is rapidly reduced under NAD(P)H consumption to the corresponding alcohol, 2-phenylethanol (I), as known from the alcoholic fermentation of *S. cerevisiae* (phenylacetaldehyde dehydrogenase, PAAD; EC 1.2.1.39). This type of conversion was demonstrated for various yeasts and is known as the EHRLICH pathway^[11,12]^. In the case of the brewer’s yeast (*S. cerevisiae*), the pathway contains at least five decarboxylases and six alcohol dehydrogenases^[13]^. 2-Phenylethanol (I) in turn can be oxygenated at the benzylic position by either a microsomal P450 monooxygenase as proposed for propyl|ethylbenzene and *Fusarium moniliforme* and *Aspergillus flavus*, respectively^[14–16]^, or by a fungal unspecific peroxygenase (UPO; EC 1.11.2.1) as shown by Kluge and coworkers^[17]^. The latter enzyme may also accomplish a second hydroxylation at the benzylic carbon (over-oxidation) directly yielding the final product 2-hydroxyacetophenone (II) via a gem-diol intermediate^[18]^. Two UPO genes were identified in the genome of *P. pouzarii*.

The second route may proceed *via* the enzymatic decarboxylation of cinnamic acid to form styrene. In this case, phenacrylate decarboxylase (PAD; EC 4.1.1.102) catalyzes the CO_2_-releasing reaction, an enzyme which is also referred to as cinnamic acid decarboxylase^[19,20]^. PADs are widely distributed among filamentous fungi and yeasts^[21,22]^ and require a prenylated flavin cofactor that is provided by a flavin prenyltransferase (EC 2.5.1.129; https://www.brenda-enzymes.org/ enzyme.php?ecno=4.1.1.102). In the next step, styrene could be oxygenated to the corresponding epoxide by a styrene monooxygenase (SMO; EC 1.14.14.11). This enzyme type is found as an NADH-reductase dependent flavoenzyme in many bacteria^[23]^; however, it has only been detected once in a fungus, namely in the eurotiomycete *Exophiala jeanselmei* (Langeron) McGinnis & A.A. Padhye^[24]^ where it apparently occurs as a cytochrome P450 protein. Fungal unspecific peroxygenase (UPO; EC 1.11.2.1) can also epoxidize styrene^[17]^. Styrene oxide can be hydrolyzed to 1-phenyl-1,2-ethandiol (III) by less specific epoxide hydrolases that exist as microsomal and soluble proteins (EC 3.3.3.9 and EC 3.3.2.10, respectively) in all eukaryotes including fungi^[25]^. Dioxygenation of the side chain of styrene by a styrene dioxygenase (SDO; EC 1.14.12.x) would directly yield 1-phenyl-1,2-ethandiol^[26]^; indications for the presence of corresponding genes have been reported in the *Candida* genome database (www.candidagenome.org/cgi-bin/GO/go.pl?goid=18567). Finally, 1-phenyl-1,2-ethandiol can be selectively dehydrogenated to 2-hydroxyacetophenone (II) by an L-glycol dehydrogenase (GlDH; EC 1.1.1.185). This enzyme was described for *Gallus gallus* (chicken), where it unspecifically oxidizes glycol derivatives with NAD(P)+ as co-substrate^[27]^. A corresponding overview is shown in Figure S1.2.

#### Methyl p-anisate

Its biosynthesis in fungi can again start from phenylalanine and proceed to 2-hydroxyacetophenone (II), as shown in figure S1.3. There are two enzymatic possibilities for the unspecific oxidation of the primary alcohol functionality of (II) into the corresponding aldehyde, either by an unspecific alcohol dehydrogenase (ADH, EC 1.1.1.1/2, dependent on NAD^+^ or NADP^+^) or by an alcohol oxidase (AOx, EC 1.2.3.1). Both enzyme types are known from yeasts and filamentous fungi^[28–30]^. Notably, AOx could deliver peroxide for subsequently acting UPOs. The phenylglyoxal formed can be oxidized by three different enzyme types: extracellular glyoxal oxidase (GlyOx, EC 1.2.3.15) present in various wood-rot fungi (Fong and Brumer 2023) or by ubiquitous unspecific aldehyde dehydrogenase (AldDH, EC 1.2.5.2, e.g. from *Aspergillus niger*, (O'Connell and Kelly 1989)) or aldehyde oxidase (AldOx, EC 1.2.3.1*,* e.g. from *Aspergillus nidulans*, (Pateman et al. 1997)) – the former and the latter again delivering peroxide. The oxidation product phenylglyoxylate (benzoylformate) is subject to decarboxylation by an eponymous decarboxylase (BFDC, EC 4.1.1.7) yielding benzaldehyde as shown for the basidiomycetous yeast *Rhodoturula graminis*^[31]^. Benzaldehyde in turn can be oxidized into benzoic acid by an NADP^+^-dependent benzaldehyde dehydrogenase (BADH, EC 1.2.1.7 as proposed for *Neurospora crassa*^[32]^ or by UPO with H_2_O_2_ as co-substrate^[33]^. Benzoic acid is specifically hydroxylated in *para*-position by benzoate 4-monooxygenase that is, in the case of *Aspergillus niger*, a P450 protein (CYP53A1^[34]^) or may be also slowly peroxygenated by UPO. The product (*p*-hydroxybenzoic acid) can be *ortho*-hydroxylated to form protocatechuate, a key intermediate of the microbial metabolism of aromatic compounds, by 4-hydroxybenzoate 4-monooxygenase (4HB4MO, EC 1.14.13.64). The latter is an NADH-dependent flavoprotein found in yeasts like *Candida* spp. and *Yarrowia lipolytica*^[35]^. The further methylation of the hydroxylated benzoic acids involves the already mentioned carboxylate transferase (CMT, for methyl ester formation) and phenol *O*-methyl transferase (for methyl ether formation) ending up the identified products (IV) and (VI), i.e. methyl *p*-methoxybenzoate (methyl anisate) and methyl 3,4-dimethoxybenzoate, respectively.


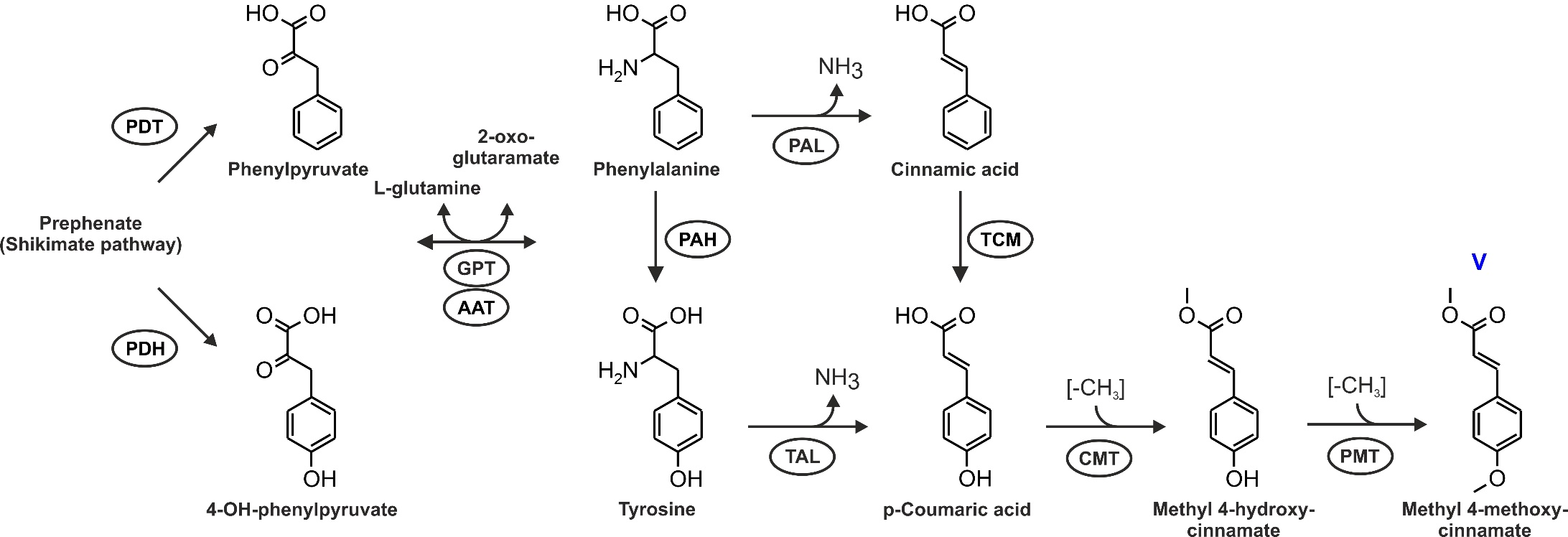


**Figure S1.1:** Proposed pathways for the fungal production of methyl *p*-methoxycinnamate derivatives detected in basidiocarp of *P. pouzarii* and surrounding wood.

AAT = Aromatic aminotransferase I (= aro8; fungal *Saccaromyces cerevisiae*; EC 2.6.1.57); CMT = Carboxyl methyl-transferase (EC 2.1.1.X); GPT = Glutamine-phenylpyruvate transaminase (EC 2.6.1.64); PAH = Phenylalanine 4-monooxygenase (EC 1.14.16.1); PAL = Phenylalanine ammonia lyase (EC 4.3.1.24); PDH = Prephenate dehydrogenase (EC 1.3.1.12); PDT = Prephenate dehydratase (EC 4.2.1.51); PMT = Phenol *O*-methyltransferase (EC 2.1.1.25); TAL = Tyrosine ammonia lyase (EC 4.3.1.23); TCM = Trans-cinnamate 4-monooxygenase (EC 1.14.14.91); blue roman numerals = detected metabolites


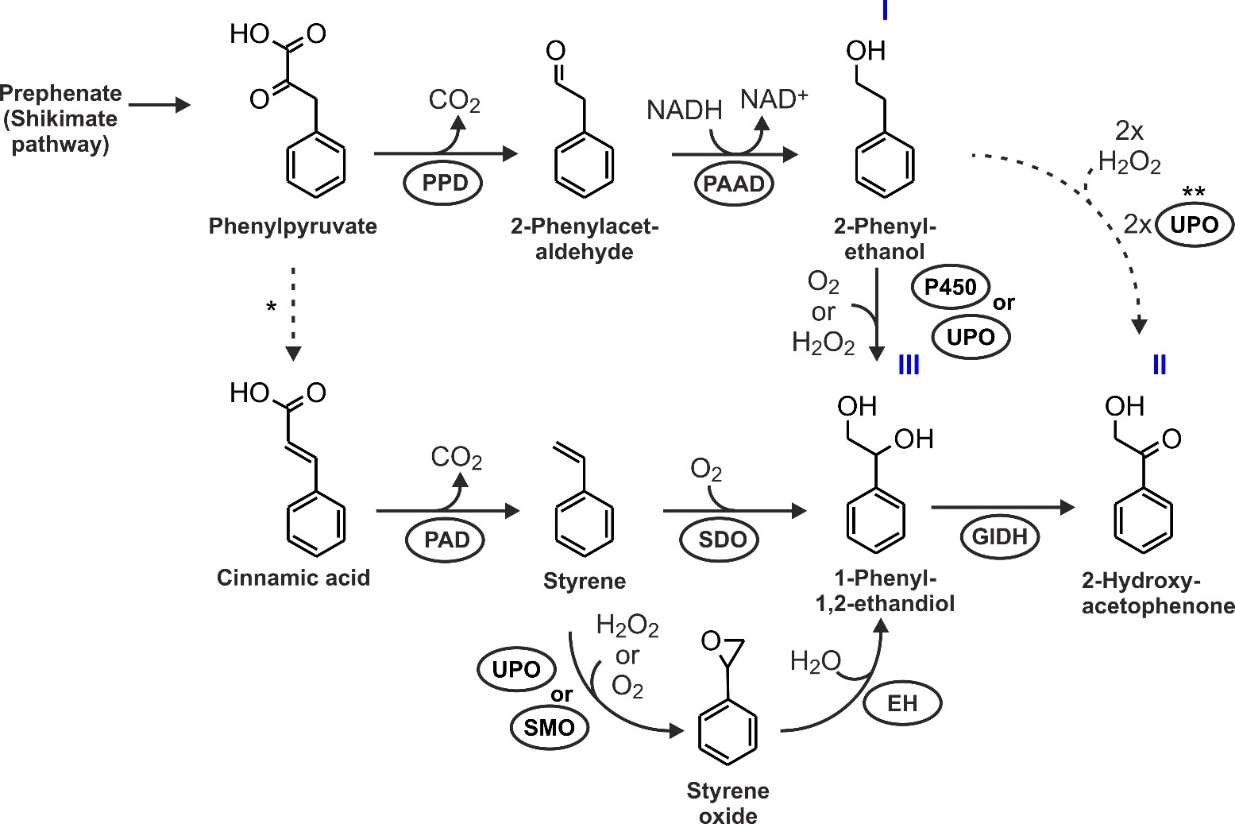


**Figure S1.2:** Proposed pathways for the fungal production of phenylethane derivatives detected in basidiocarp of *P. pouzarii* and surrounding wood.

EH = Epoxide hydrolase (EC 3.3.2.9 - microsomal; EC 3.3.2.10 - soluble); GlDH = L-Glycol dehydroganase (EC 1.1.1.185); P450 = microsomal unspecific monooxygenase (EC 1.14.14.1); PAAD = Phenylacetaldehyde dehydrogenase (EC 1.2.1.39); PAD = Phenacrylate decarboxylase (EC 4.1.1.102); PPD = Phenylpyruvate decarboxylase (EC 4.1.1.43); SDO = Styrene dioxygenase (EC 1.14.12.x); SMO = Styrene monooxygenase (SMO; EC 1.14.14.11); UPO = unspecific peroxygenase (EC 1.11.2.1); blue roman numerals = detected metabolites; * described for methyl *p*-methoxycinnamate pathway, compare above; ** *via* geminal diol


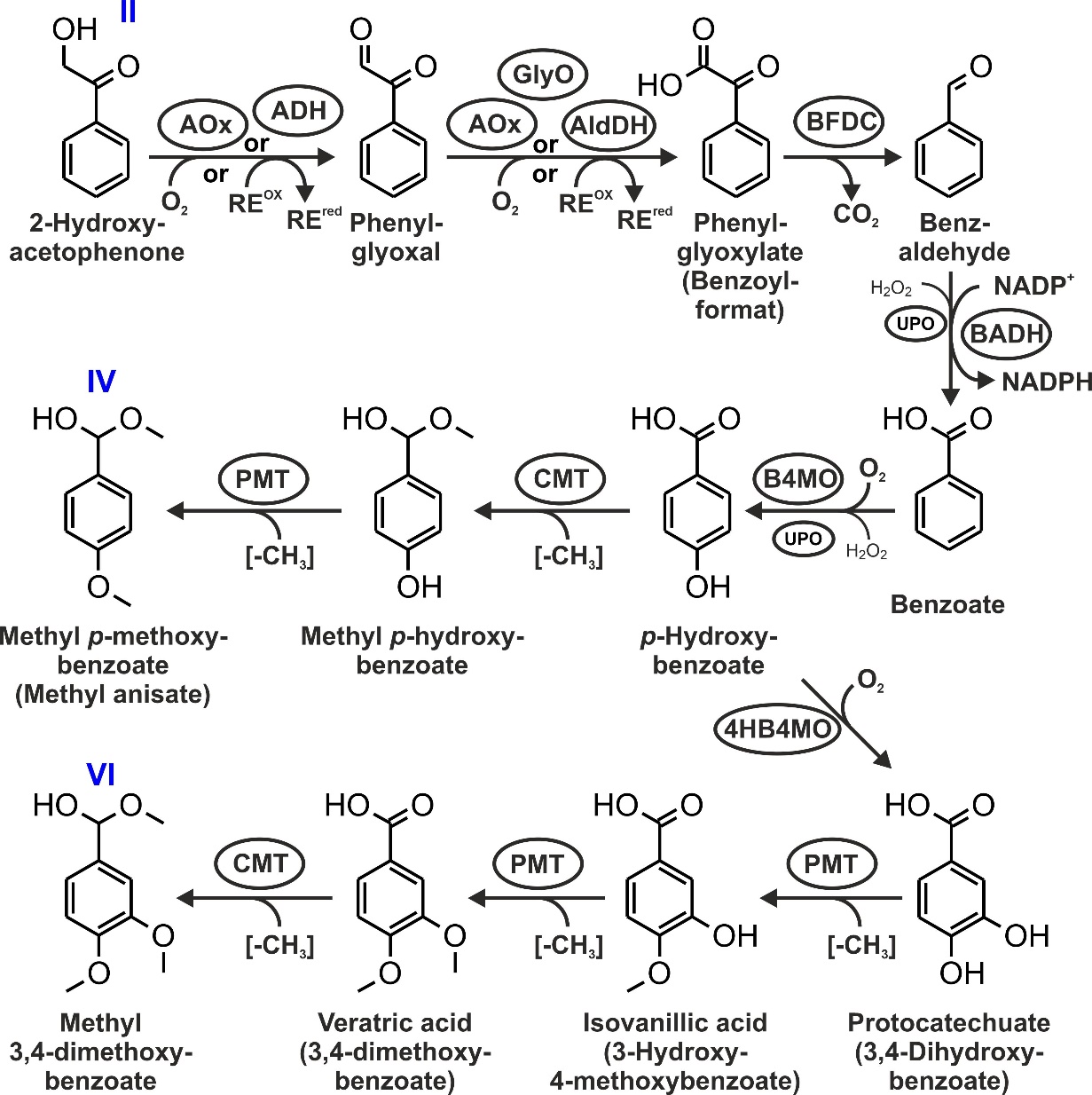


**Figure S1.3:** Proposed pathways for the fungal production of methyl anisate and methyl veratrate detected in basidiocarp of *P. pouzarii* and surrounding wood. ADH = Unspecific alcohol dehydrogenase (EC 1.1.1.1/2); AldDH = Unspecific aldehyde dehydrogenase (EC 1.2.5.2); AldO = Unspecific aldehyde oxidase (EC 1.2.3.1); AOx = Alcohol oxidase, EC 1.1.3.13); BADH = Benzaldehyde dehydrogenase (EC 1.2.1.7); BFDC = Benzoylformate decarboxylase (EC 4.1.1.7); B4MO = Benzoate 4-monooxygenase (P450, EC 1.14.14.92); CMT = Carboxylate methyl transferase (EC 2.1.1.x); GlyO = Glyoxal oxidase (EC 1.2.3.15); UPO = Unspecific peroxygenase (EC 1.11.2.1); PMT = Phenol O-methyltransferase (EC 2.1.1.25); 4HB4MO = 4-Hydroxybenzoate 4-monooxygenase (EC 1.14.14.64); RE - Reducing equivalent - oxidized or reduced; Blue roman numerals = detected metabolites

**Table S1.4:** Homologous fragrance producing genes found in *P. pouzarii*.

| **Enzyme name** | | **EC** | | **Reference used** | | **Homologous gene** | **% similarity** |
| --- | --- | --- | --- | --- | --- | --- | --- |
| ***Methyl p-methoxycinnamate*** | | | | | | | |
| glutamate-prephenate aminotransferase (PAT) | | EC 2.6.1.79 | | 5WMH_A | | THH12218 | 30.6 |
| prephenate dehydrogenase (PDH) | | EC 1.3.1.12 | | 3B1F_A | | - | - |
| prephenate dehydratase (PDT) | | EC 4.2.1.51 | | 4LUB_A | | THH11120 | 29.1 |
| glutamine-phenylpyruvate transaminase (GPT) | | EC 2.6.1.64 | | 1YIY_A | | THH12218 | 40.2 |
| aromatic aminotransferase (AAT) | | EC 2.6.1.57 | | 4JE5_A | | THH05713 | 34.2 |
| phenylalanine 4-monooxygenase (PAH) | | EC 1.14.16.1 | | 5JK6_A | | - | - |
| phenylalanine ammonia lyase (PAL) | | EC 4.3.1.24 | | 3NZ4_A | | THH08002 | 35.1 |
| *trans*-cinnamic acid 4-monooxygenase (TCM) | | EC 1.14.14.91 | | QOJ43647 | | THH09410 | 63 |
| tyrosine ammonia lyase (TAL) | | EC 4.3.1.23 | | CAF1733460 | | THH08002 | 25.6 |
| carboxylmethyl transferase (CMT) | | EC 2.1.1.x | | e.g. GJE94453 | | THH06496 | 31.5 |
| phenolic *O*-methyltransferase (PMT) | | EC 2.1.1.25 | | no reference available | |  |  |
| ***Phenylethane derivatives*** | | | | | | | |
| phenylpyruvate decarboxylase (PPD) | | EC 4.1.1.43 | | 2NXW | | THH09531 | 27.6 |
| phenylacetaldehyde dehydrogenase (PAAD) | | EC 1.2.1.39 | | 4O5H | | THH04684 | 51.7 |
| unspecific peroxygenase (UPO) | | EC 1.11.2.1 | | 5FUJ | | THH07130 | 31.7 |
| phenacrylate decarboxylase (PAD) | | EC 4.1.1.102 | | 6EVA | | - | - |
| flavin prenyltransferase | | EC 2.5.1.129 | | 6QLG | | - | - |
| styrene monooxygenase (SMO) | | EC 1.14.14.11 | | WZI45026 | | - | - |
| styrene dioxygenase (SDO) | | EC 1.14.12.x | | e.g. 4HM7 | | THH05920 | 35.2 (only subdomain) |
| L-glycol dehadrogenase (GDH) | | EC 1.1.1.185 | | no reference available | |  |  |
| ***Methyl p-anisate*** | | | | | | | |
| alcohol dehydrogenase (ADH) | | EC 1.1.1.1 | | 5ENV | | THG94286 | 47.4 |
| alcohol oxidase (AOx) | | EC 1.2.3.1 | |  | | several AA3 enzymes  (table 1 in main text) |  |
| glyoxal oxidase (GlyO) | | EC 1.2.3.15 | | WFD37613 | | THH04913 | 45 |
| aldehyde dehydrogenase (AldDH) | | EC 1.2.5.2 | | ABG51795 | | THG98060 | 54.1 |
| eponymous decarboxylase (BFDC) | | EC 4.1.1.7 | | 1BFD | | THH12005 | 26.5 |
| benzaldehyde dehydrogenase (BADH) | | EC 1.2.1.7 | | 5UCD | | THH03861 | 33.3 |
| 4-hydroxybenzoate 4-monooxygenase (4HB4MO) | | EC 1.14.13.64 | | no reference available | |  |  |
| glutamate-prephenate aminotransferase | EC 2.6.1.79 | | 5WMH_A | | THH12218 | | 30.6 |
| prephenate dehydrogenase | EC 1.3.1.12 | | 3B1F_A | | - | | - |
| prephenate dehydratase | EC 4.2.1.51 | | 4LUB_A | | THH11120 | | 29.1 |
| glutamine-phenylpyruvate transaminase | EC 2.6.1.64 | | 1YIY_A | | THH12218 | | 40.2 |
| aromatic aminotransferase | EC 2.6.1.57 | | 4JE5_A | | THH05713 | | 34.2 |
| phenylalanine 4-monooxygenase | EC 1.14.16.1 | | 5JK6_A | | - | | - |
| phenylalanine ammonia lyase | EC 4.3.1.24 | | 3NZ4_A | | THH08002 | | 35.1 |
| *trans*-cinnamic acid 4-monooxygenase | EC 1.14.14.91 | | QOJ43647 | | THH09410 | | 63 |
| tyrosine ammonia lyase | EC 4.3.1.23 | | CAF1733460 | | THH08002 | | 25.6 |
| carboxylmethyl transferase | EC 2.1.1.x | |  | | many available | |  |
| phenolic *O*-methyltransferase | EC 2.1.1.25 | | no reference available | |  | |  |

## S2: PCR protocol

PCR was performed in 25 µl reactions containing 12.5µl Dream Taq MasterMix (ThermoFisher Scientific, Darmstadt, Germany), 1 µl of each primer (10 pmol) and ~ 20ng template DNA. The following cycling conditions were used for target PCR of bacterial and fungal marker regions.

**Table S2.1:** PCR protocols used in this study

| **PCR step** | **Fungi** | | | **Bacteria** | | |
| --- | --- | --- | --- | --- | --- | --- |
|  | Temp | Duration | Cycles | Temp | Duration | Cycles |
| **Initial denaturation** | 95°C | 5 min | 35 | 94°C | 3 min | 32 |
| **Denaturation** | 95°C | 5 min |  | 94°C | 0:45 min |  |
| **Annealing** | 55°C | 1 min |  | 50°C | 1 min |  |
| **Elongation** | 72°C | 1:30 min |  | 72°C | 1:30 min |  |
| **Final elongation** | 72°C | 10 min |  | 72°C | 10 min |  |

## S3: Figures


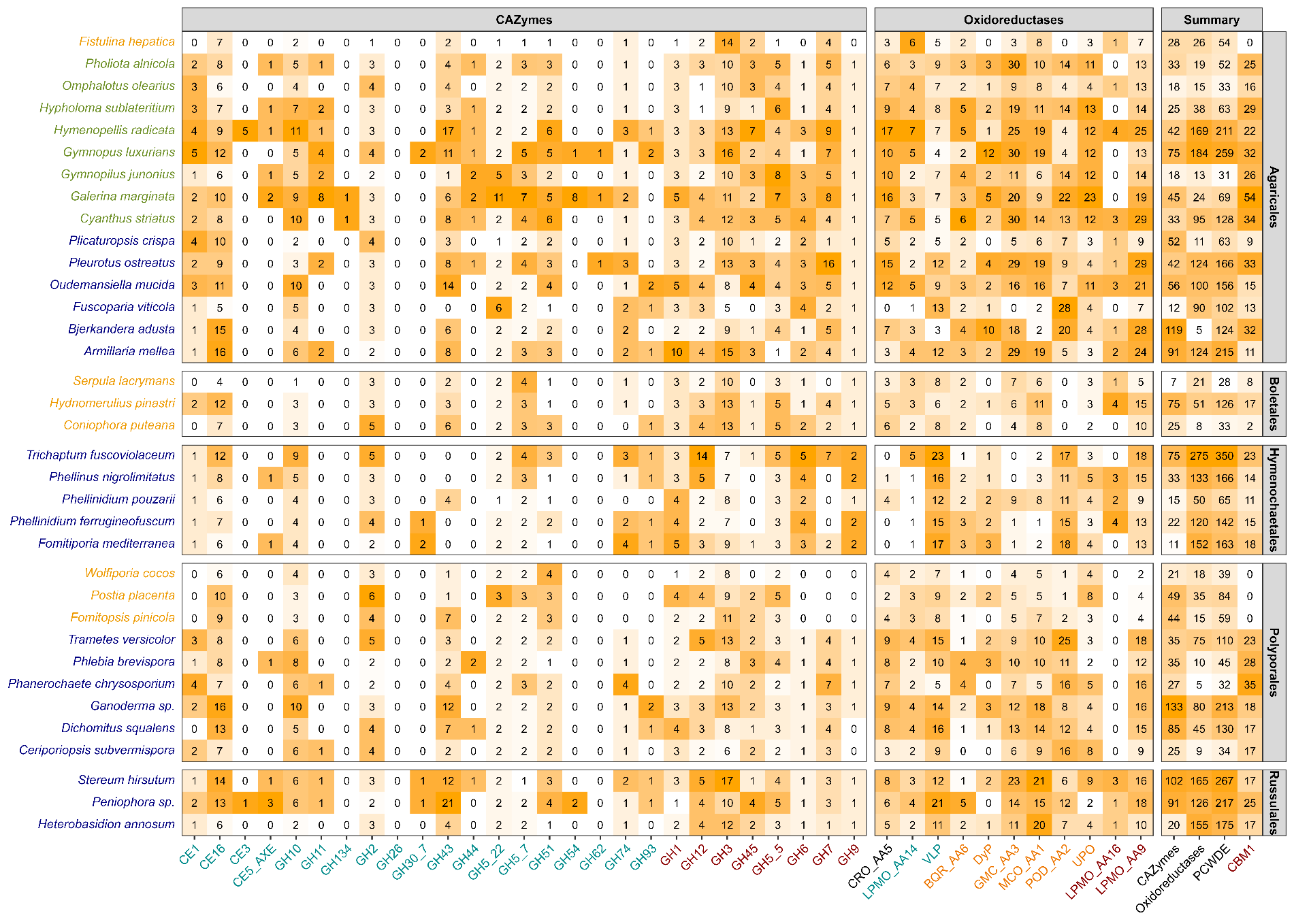


**Figure S3.1:** Heatmap showing gene numbers for plant cell-wall degrading enzymes (PCWDE) and cellulose-binding modules (CBM1) in genomes of several wood-degrading fungi (orange = high numbers, white = low numbers). Species (y-axis) are colored according to their lifestyle (green = decayed wood, blue = white rot, orange = brown rot) and enzymes (x-axis) are colored depending on their respective substrate (teal = hemicellulose, red = cellulose, orange = lignin). Note that CRO (AA5) can act on lignin and cellulose. Summary contains total number of CAZymes, total number of oxidoreductases and total number of PCWDE (CAZymes + oxidoreductases). *Hymenochaetales* data were supplemented using MycoCosm data: *Fomitiporia mediterranea*^[36]^; *Phellinus ferrogineofuscum* – JGI: SpKPhefer14 v1.0^[37]^; *Phellinus nigrolimitatus*^[38]^; *Trichaptum fuscoviolaceum*: GenBank: CAJSYZ000000000^[37]^


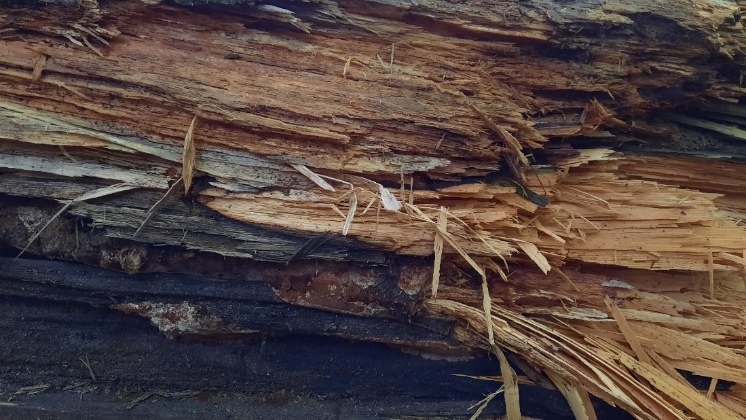

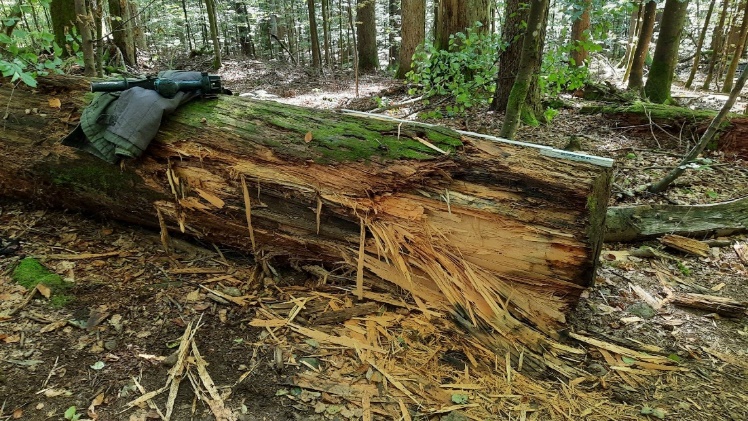


**Figure S3.2:** A splintered log of *Abies alba* (TA19, estimated decay class 3 or 4) on which we found several fruit bodies of *Phellinidium pouzarii* (arrows), after the outer layer (bark and sapwood) had been partially destroyed by an unknown incident. Photo: Harald Kellner

**
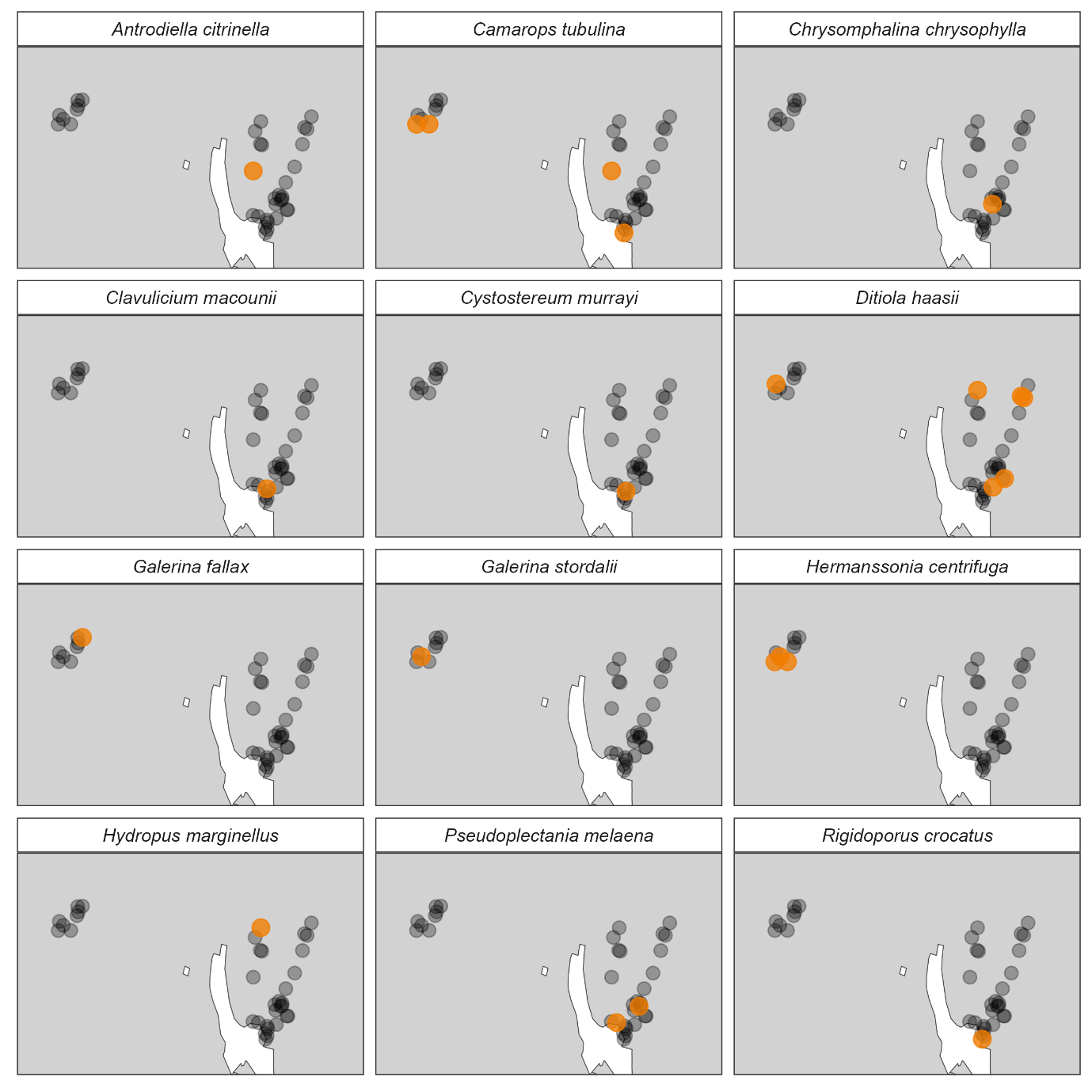
Figure S3.3:** Maps indicating the position other rare fungal species detected in Watzlik-Hain (left) and Mittelsteighütte (right) in 2022 using amplicon sequencing (grey: forest area, white: arable land/settlement).

**
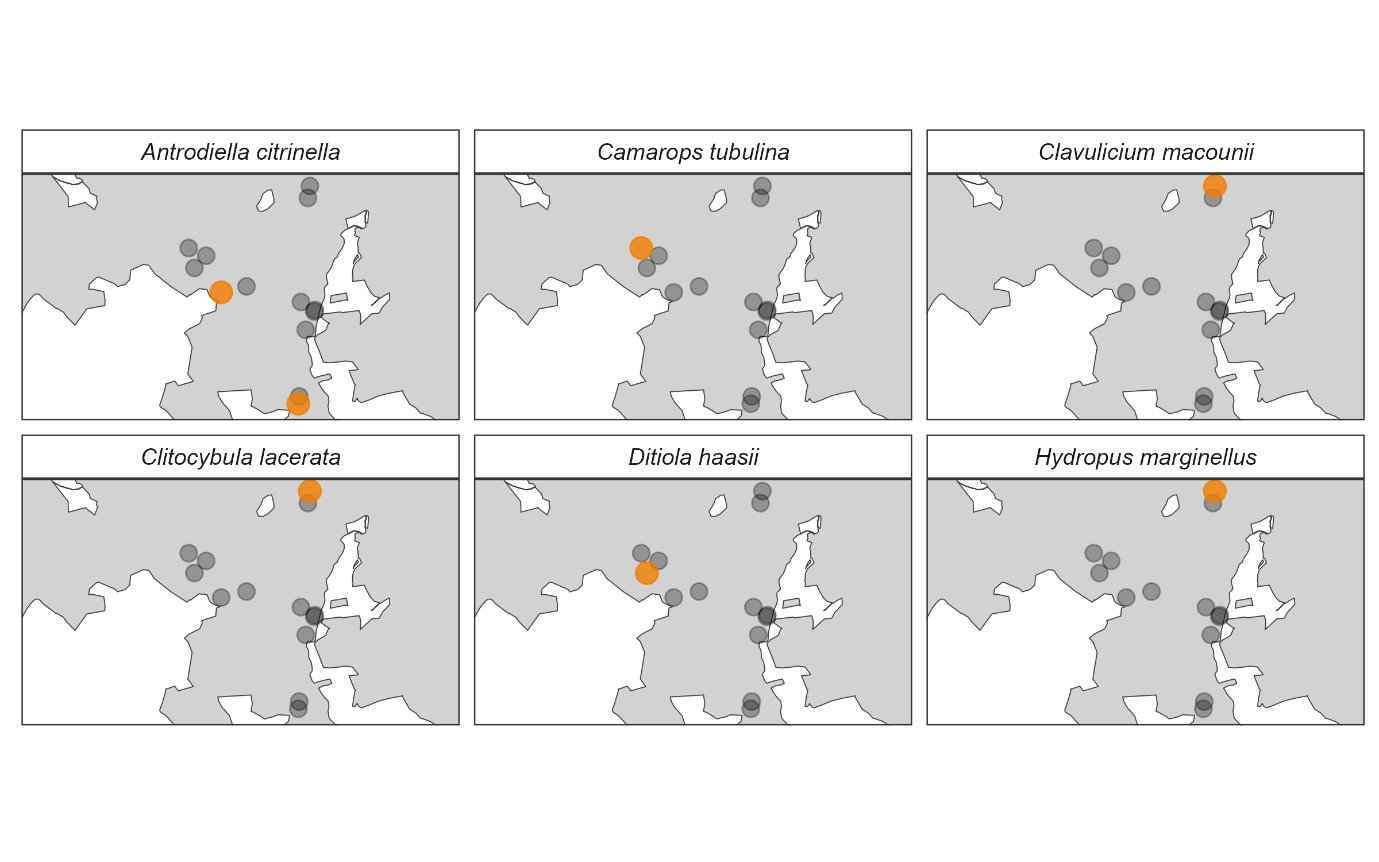
Figure S3.4:** Maps showing the locations of rare fungal species detected by amplicon sequencing (2022 data set) in the Eisenmannhaus region.


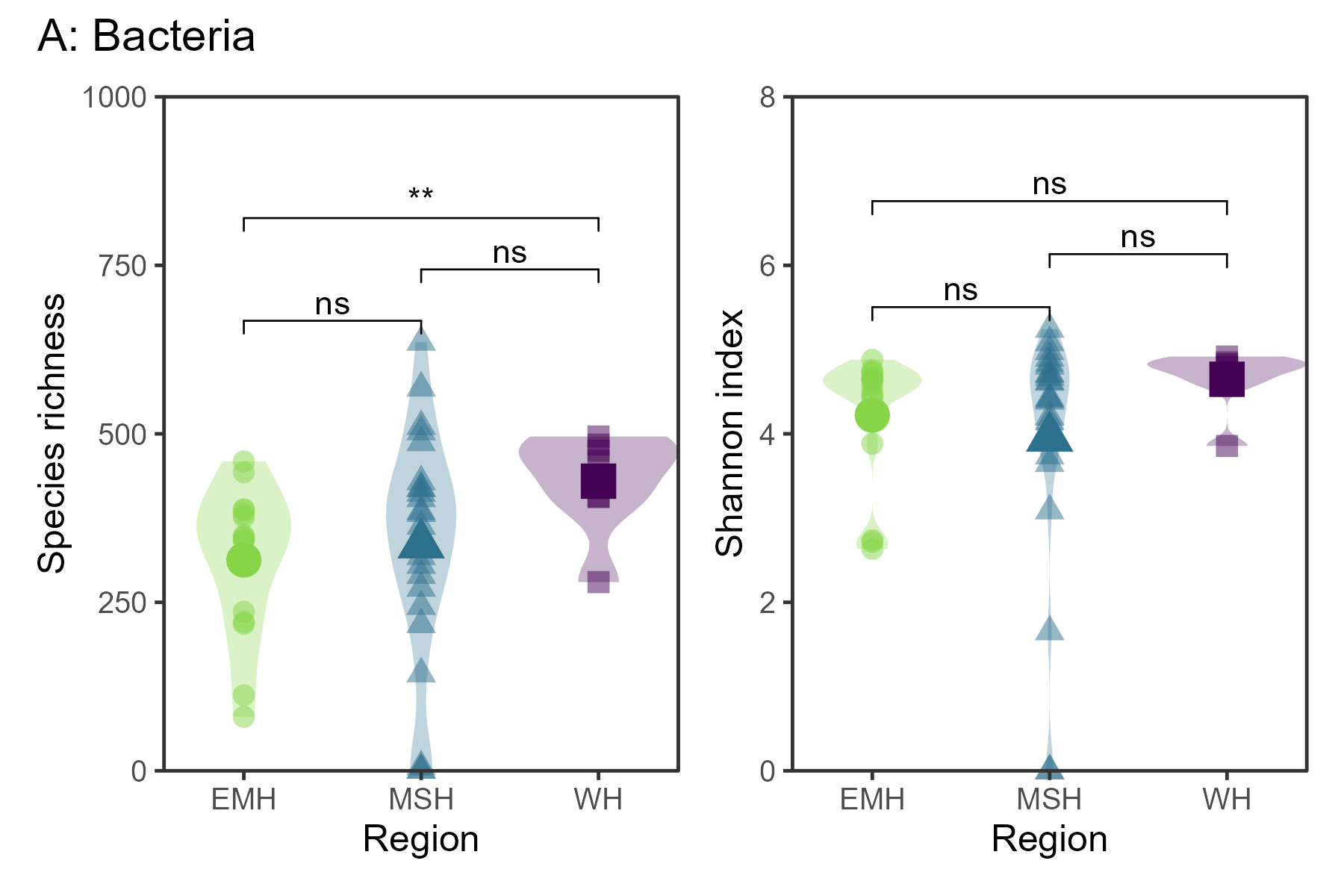

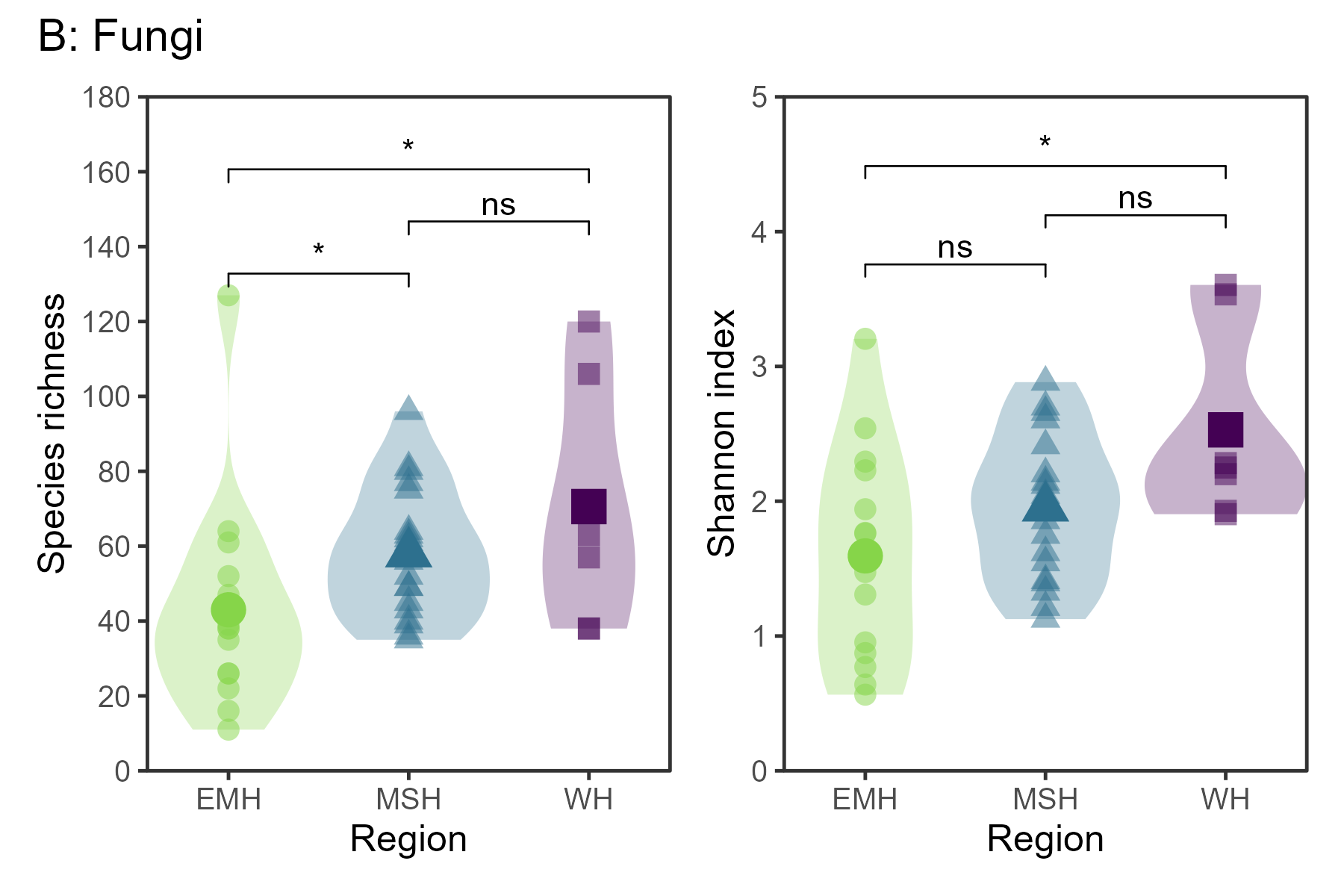


**Figure S3.5:** *Alpha* diversity measures of bacterial (A) and fungal communities (B) (2022 samples) across three sampled regions; parentheses display the results of Wilcoxon tests (ns: not significant, *: p < 0.05, **: p < 0.01, ***: p < 0.001), larger solid dots: mean.


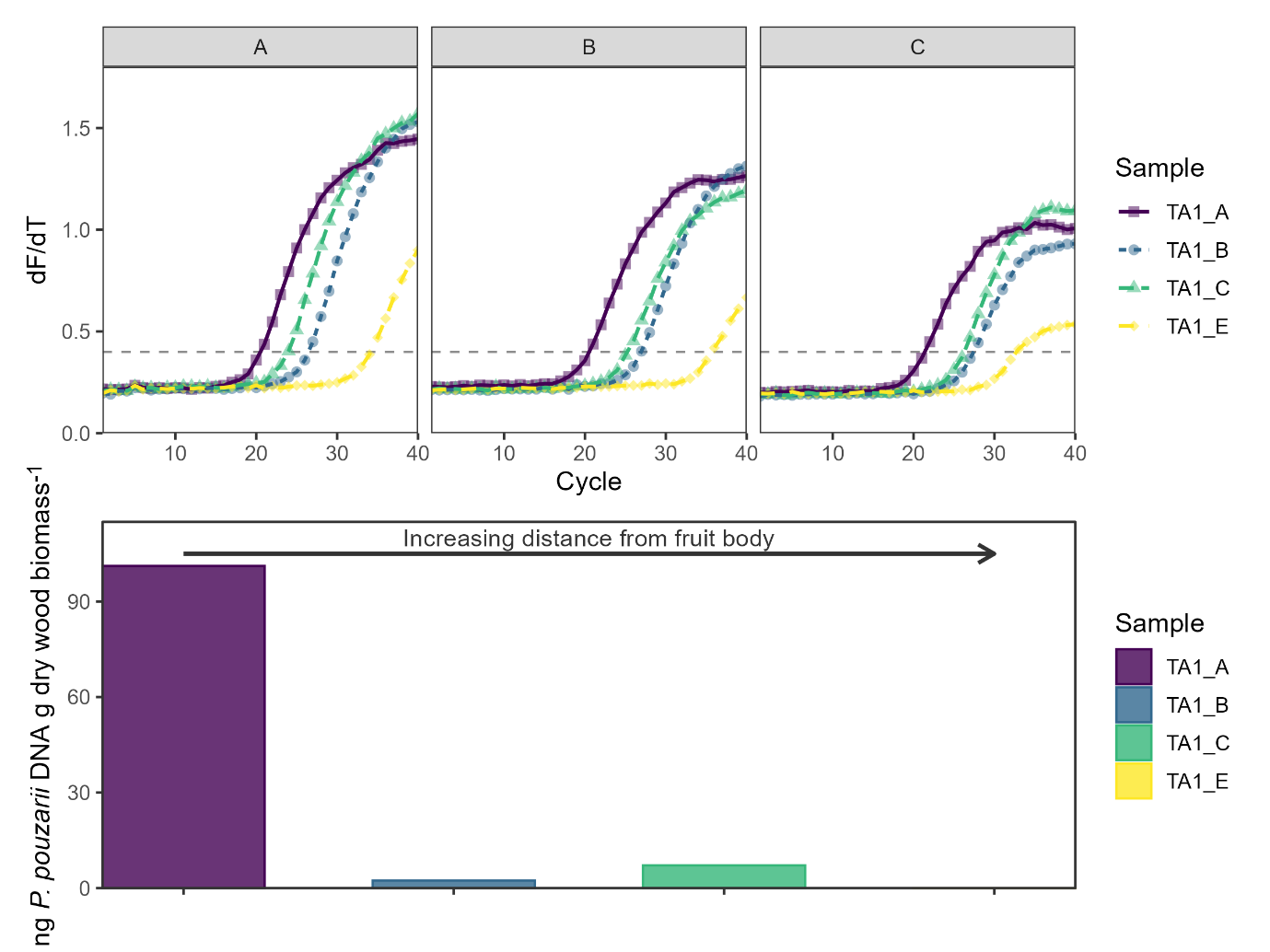


**Figure S3.6**: qPCR results for repeated sampling of TA1 (2022) with increasing distance (1 m steps) from the fruit bodies. Top panel: Change in fluorescence (dF) per change in temperature (dT) across qPCR cycles across replicates (A-C). Bottom panel: Amount of *P. pouzarii* DNA as verified by qPCR in samples taken in increasing distance from a fruit body.


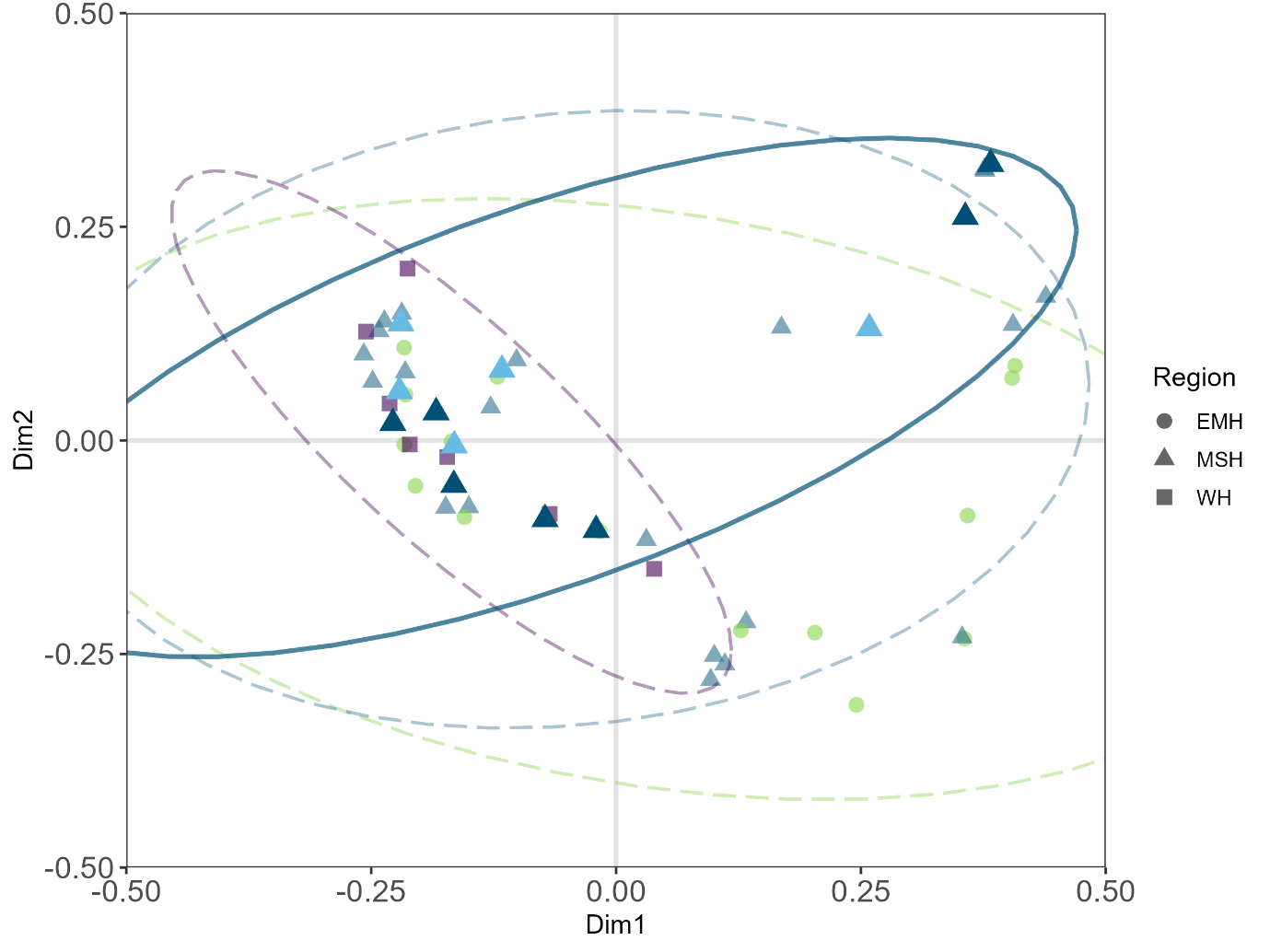


**Figure S3.7:** Principal Coordinates Analysis of bacterial communities highlighting samples in which *P. pouzarii* was detected using amplicon sequencing in 2022 (light blue) and 2021 (dark blue). Transparent symbols indicate samples where we did not detect *P. pouzarii* using amplicon sequencing or qPCR (green circle - EMH: Eisenmannhaus, bluegrey triangle - MSH: Mittelsteighütte, purple rectangle -WH: Watzlik-Hain). Solid triangles represent *P. pouzarii* positive samples (exclusively from Mittelsteighütte as we did not detect it anywhere else) from 2021 (dark blue solid triangles) and 2022 (light blue solid triangles).


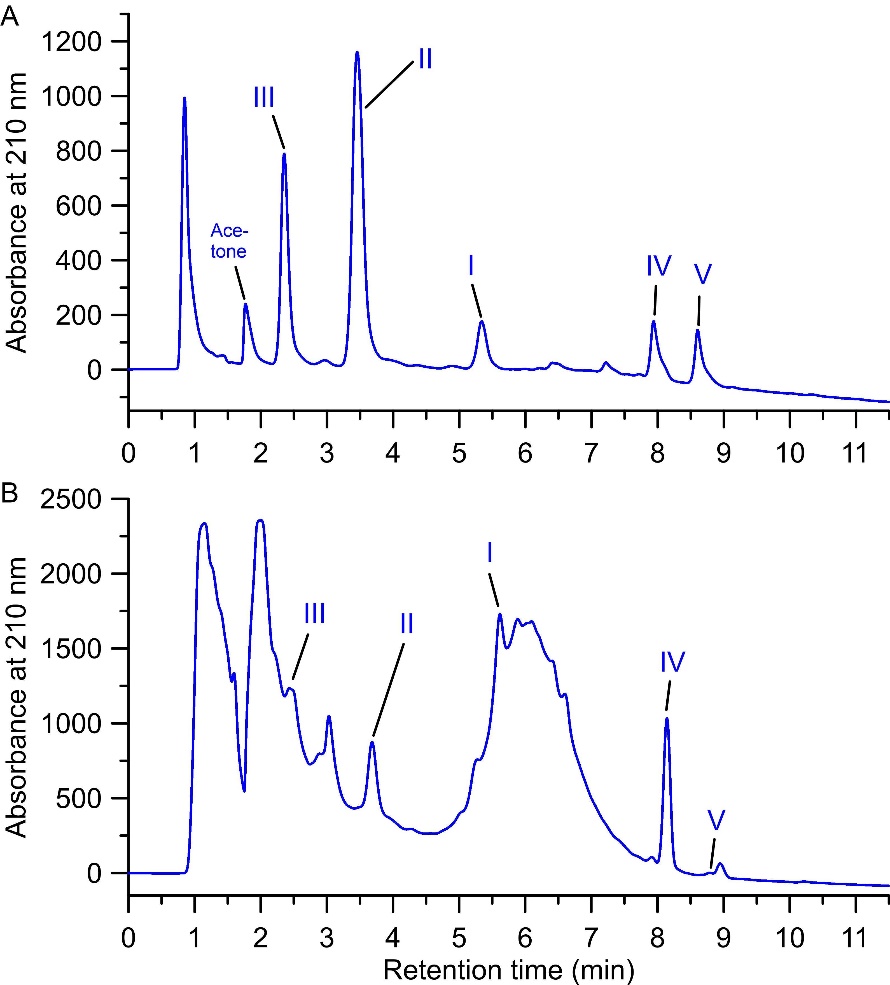


**Figure S3.8a:** HPLC-DAD elution profiles of the acetone extract from a fruit body of *P*. *pouzarii* from log TA19 (**A**), injected as 1:1-diluted sample in A. dest., and from splintered wood of log TA19 (**B**), injected as 1:5-diluted sample in A. dest. (**I**) 2-phenylethanol, (**II**) 2-hydroxyacetophenone, (**III**) 1-phenyl-1,2-ethandiol, (**IV**) methyl *p*-anisate, (**V**) methyl 4-methoxycinnamate.


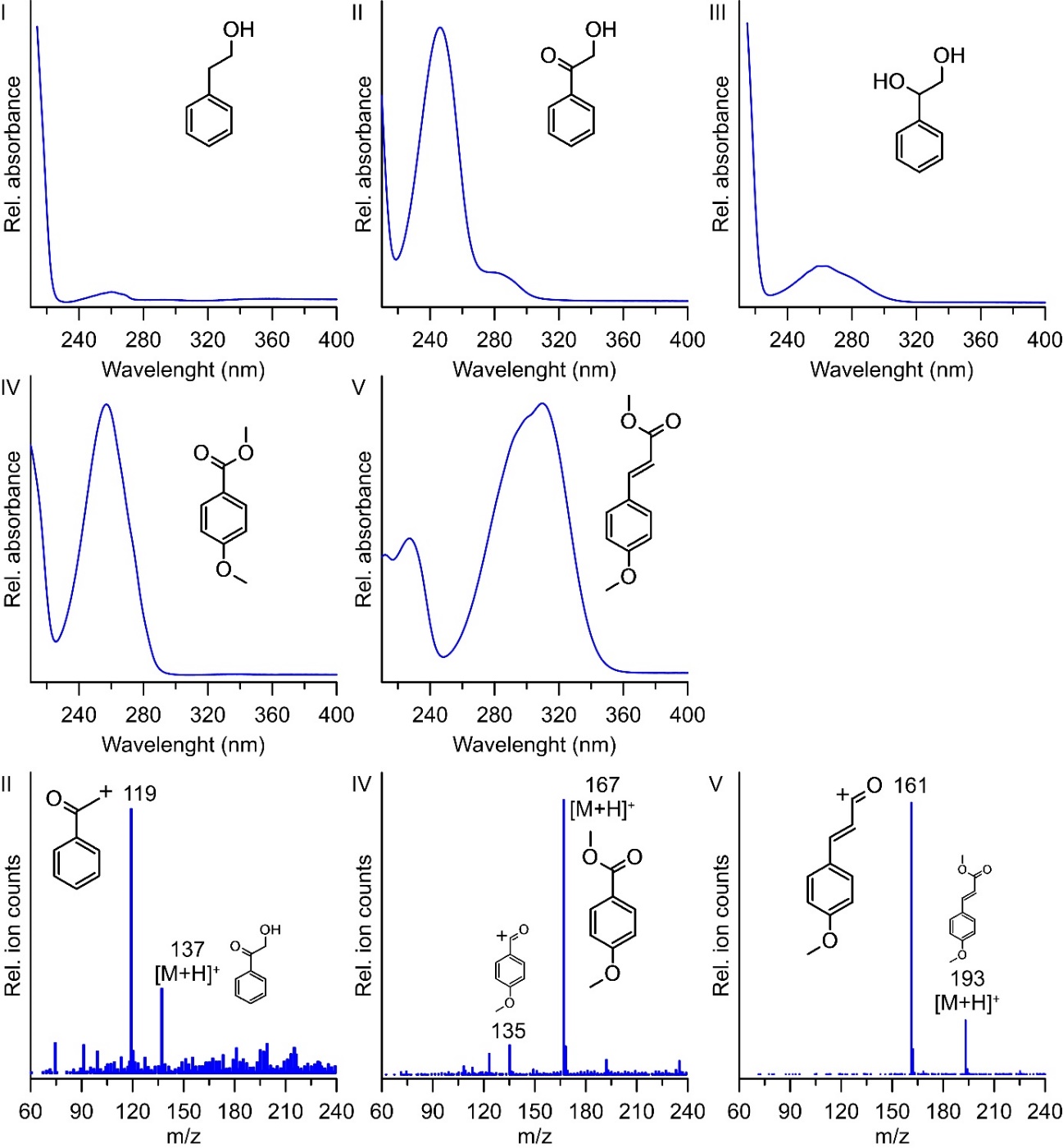


**Figure S3.8b:** UV-Vis and mass spectra recorded in the course of HPLC-DAD-MS analysis of liquid extracts of a fruit body of *P*. *pouzarii* (from log TA19) and from splintered wood of log TA19; numbering according to the legend of Figure S3.8a.


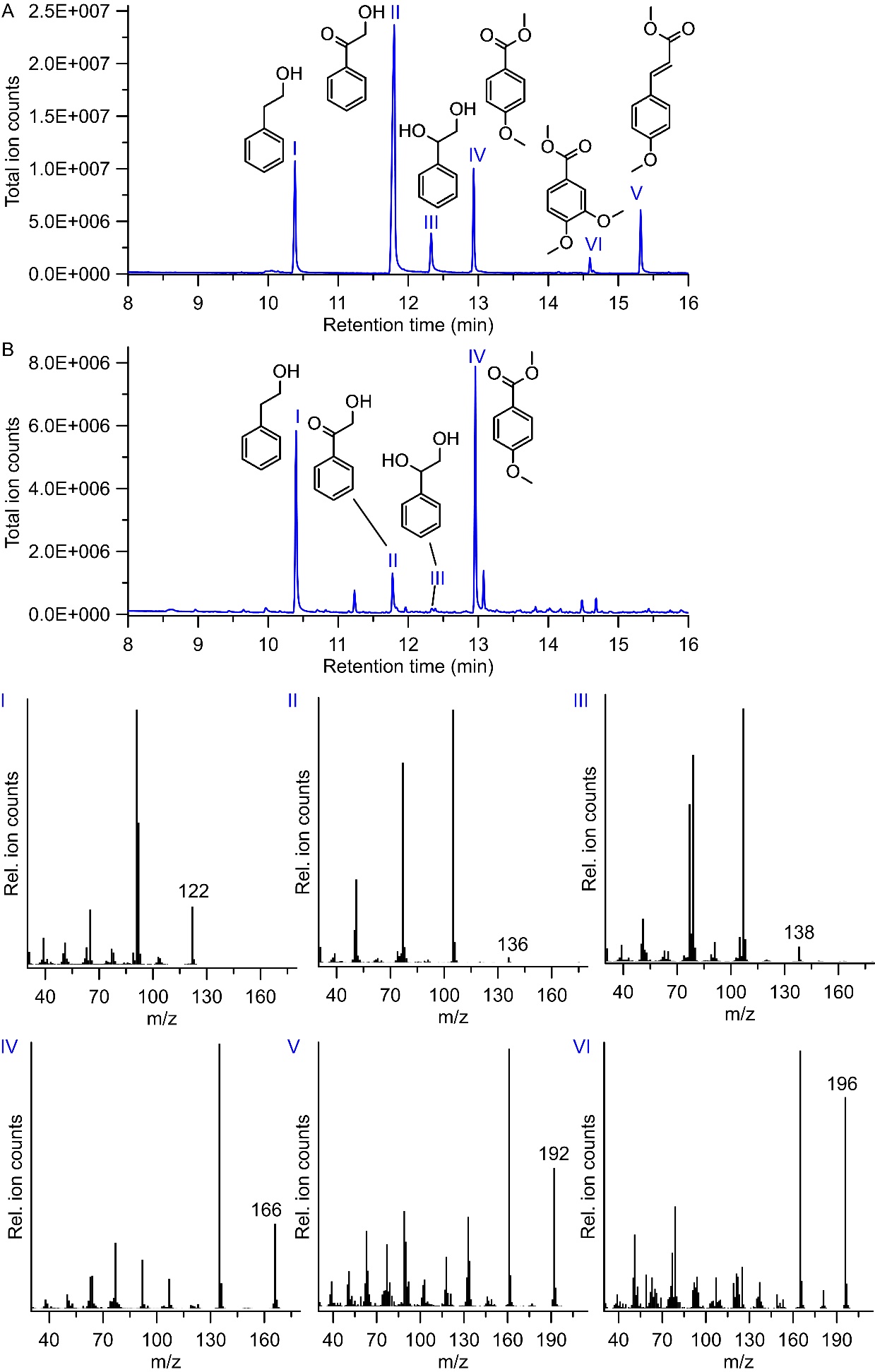


**Figure S3.8c**: GC-MS elution profiles of liquid dichloromethane extracts of **A**) a fruit body of *P*. *pouzarii* (log TA19), and **B**) from splintered wood of log TA19. I-VI: Mass spectra of the corresponding aromatic compounds identified in the chromatograms: (I) 2-phenylethanol, (II) 2-hydroxyacetophenone, (III) 1-phenyl-1,2-ethandiol, (IV) methyl p-anisate, (V) methyl 4-methoxycinnamate, (VI) methyl 3,4-dimethoxybenzoate.


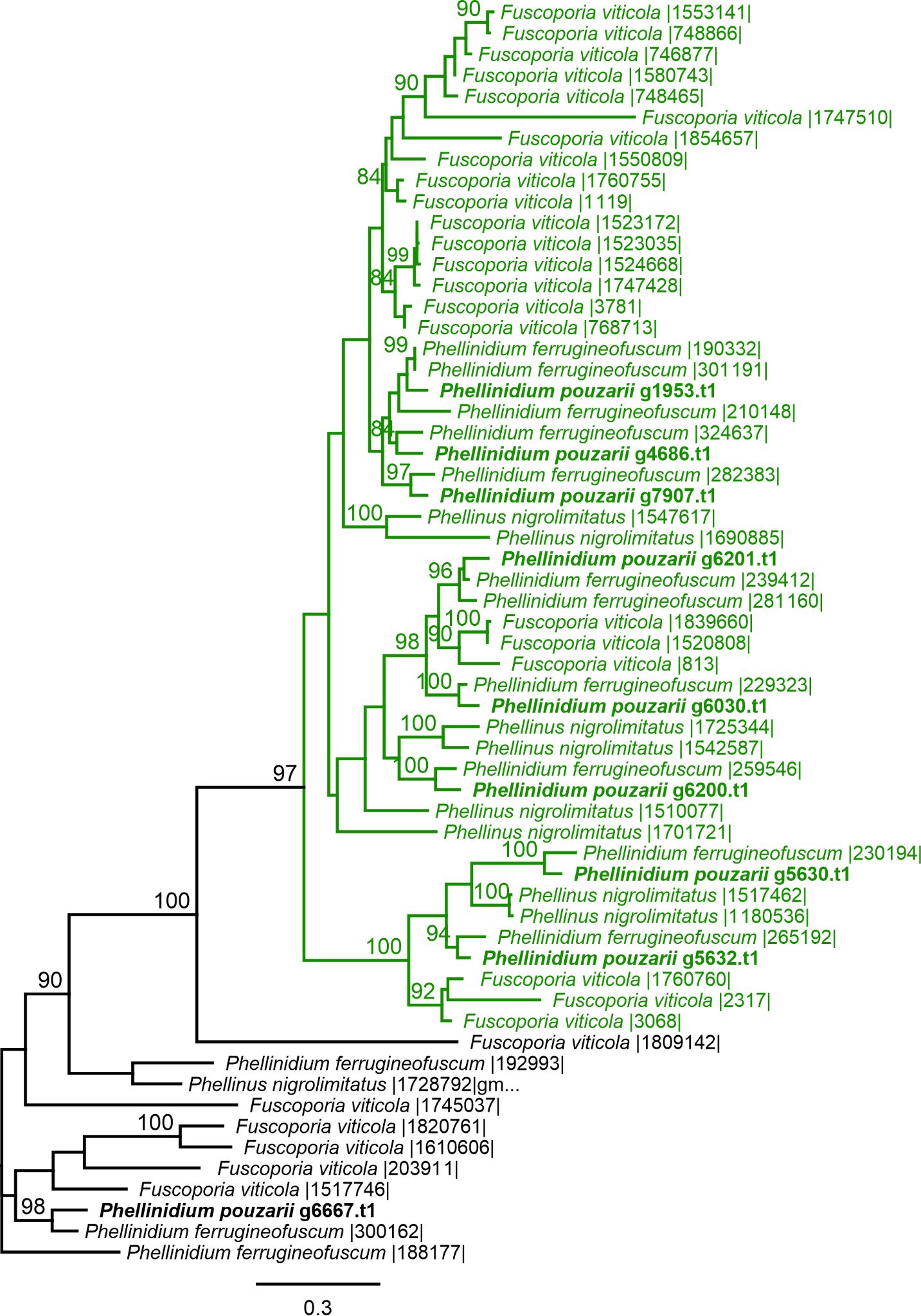


**Figure S3.9**: Maximum likelihood tree of hymenomycetous manganese peroxidases (MnPs) using RAxML and 500 bootstrapping replicates indicated above branches. In green, all MnP sequences of the long type containing an additional disulfide bridge at the C-terminal end, and in black all short MnPs. Numbers after the species name indicate the accession number of JGI, but in contrast to the latter database, the currently accepted scientific names are used (instead of sometimes confusing abbreviations). At JGI: Phevit1 - *Fuscoporia* *viticola*, Pheni1 - *Phellinus* *nigrolimitatus*, Phefer1 - *Phellinidium* *ferrugineofuscum*.


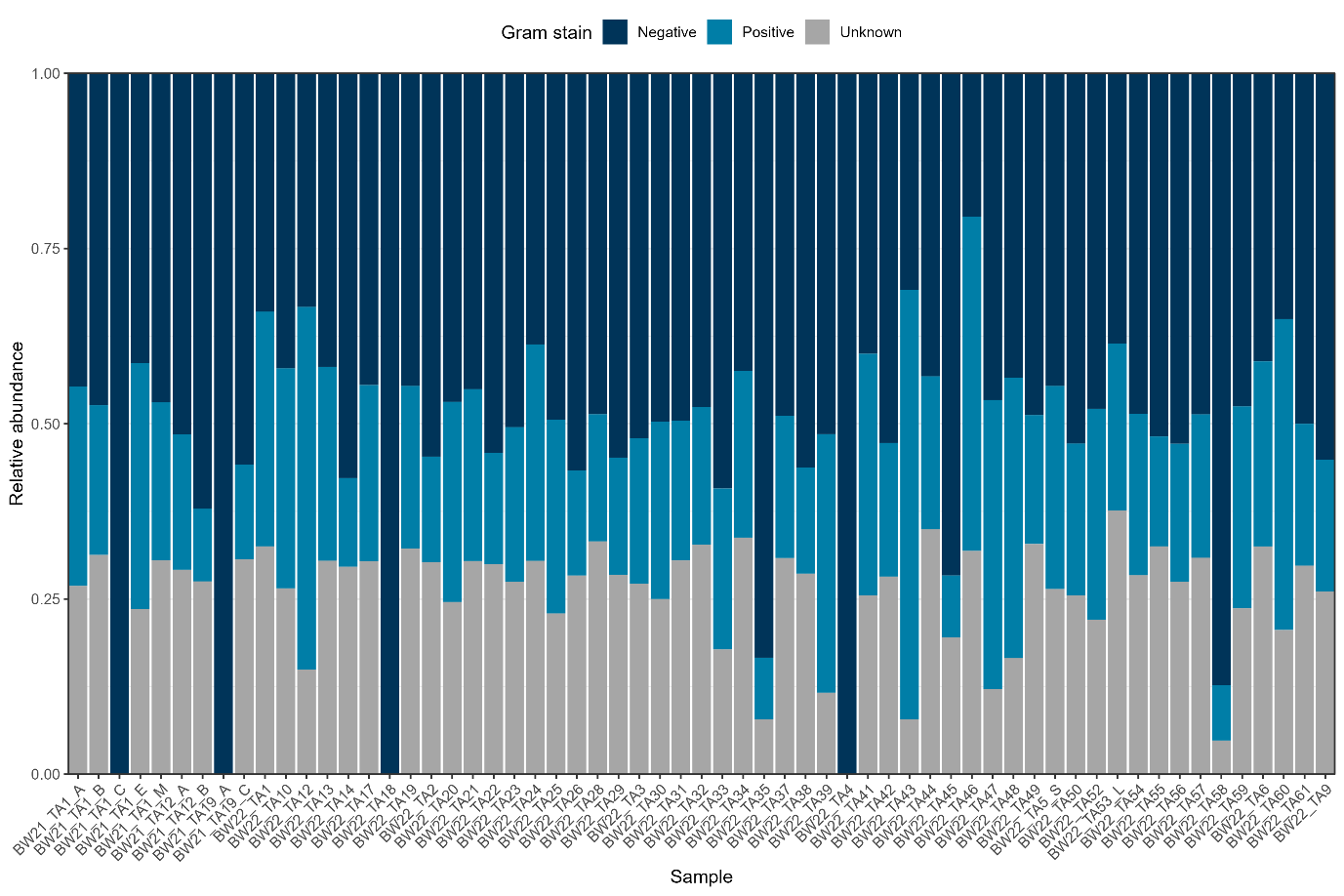


**Figure S3.10:** Relative abundances of gram-negative and positive bacterial OTUs in *Abies alba* deadwood (2021 and 2022 samples).

**
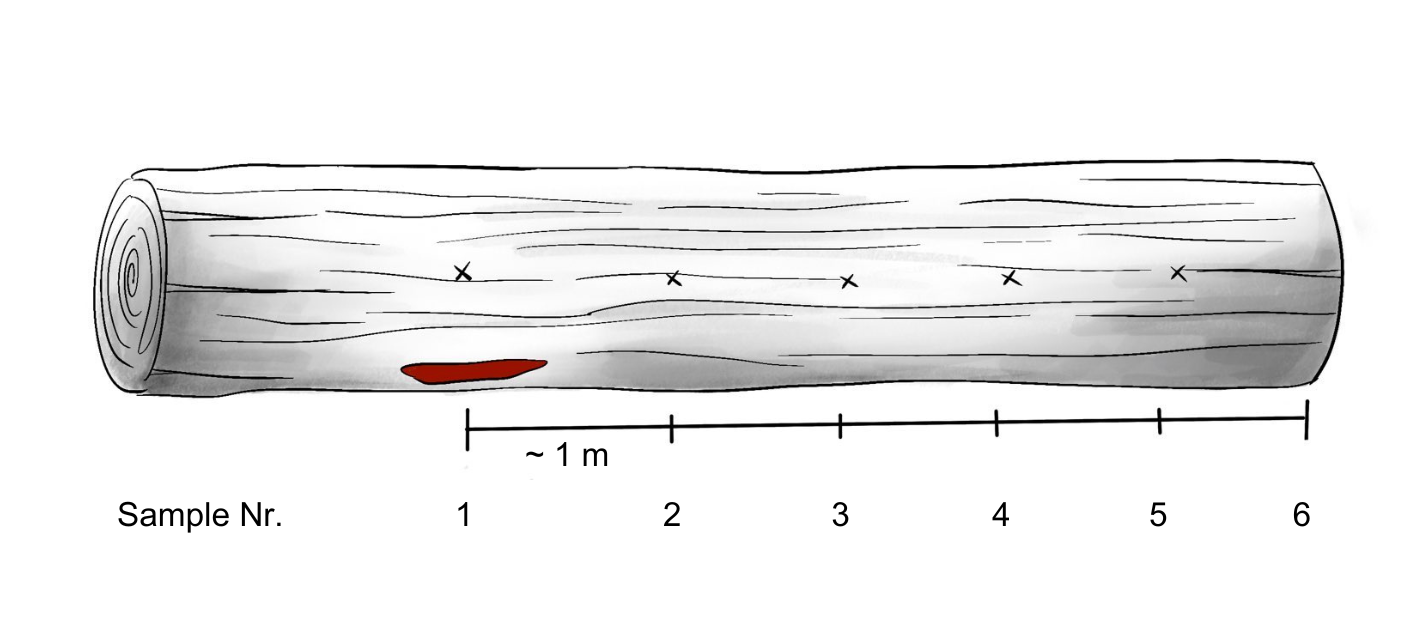
Figure S3.11:** Example of the sampling scheme for TA1, TA12 and TA19 in 2021; red mark indicates position of the *P. pouzarii* fruit body, x marks indicate drilling positions and their according labels.

**Figure S3.12:** Principal Components Analysis (PCA) of fungal plant cell-wall degrading enzymes (D) additionally divided according to their substrate (A: Cellulose-degrading enzymes; B: Hemicellulose-degrading enzymes, C: Lignin-degrading enzymes, with the position of *P. pouzarii* highlighted; species belonging to the order *Hymenochaetales* are also labelled with their scientific name. Colors and ellipses indicate fungal lifestyle: blue, solid line – brown rot, yellow – decayed wood (not enough points for ellipse), red, dashed line – white rot. First two axes of the PCA are displayed.


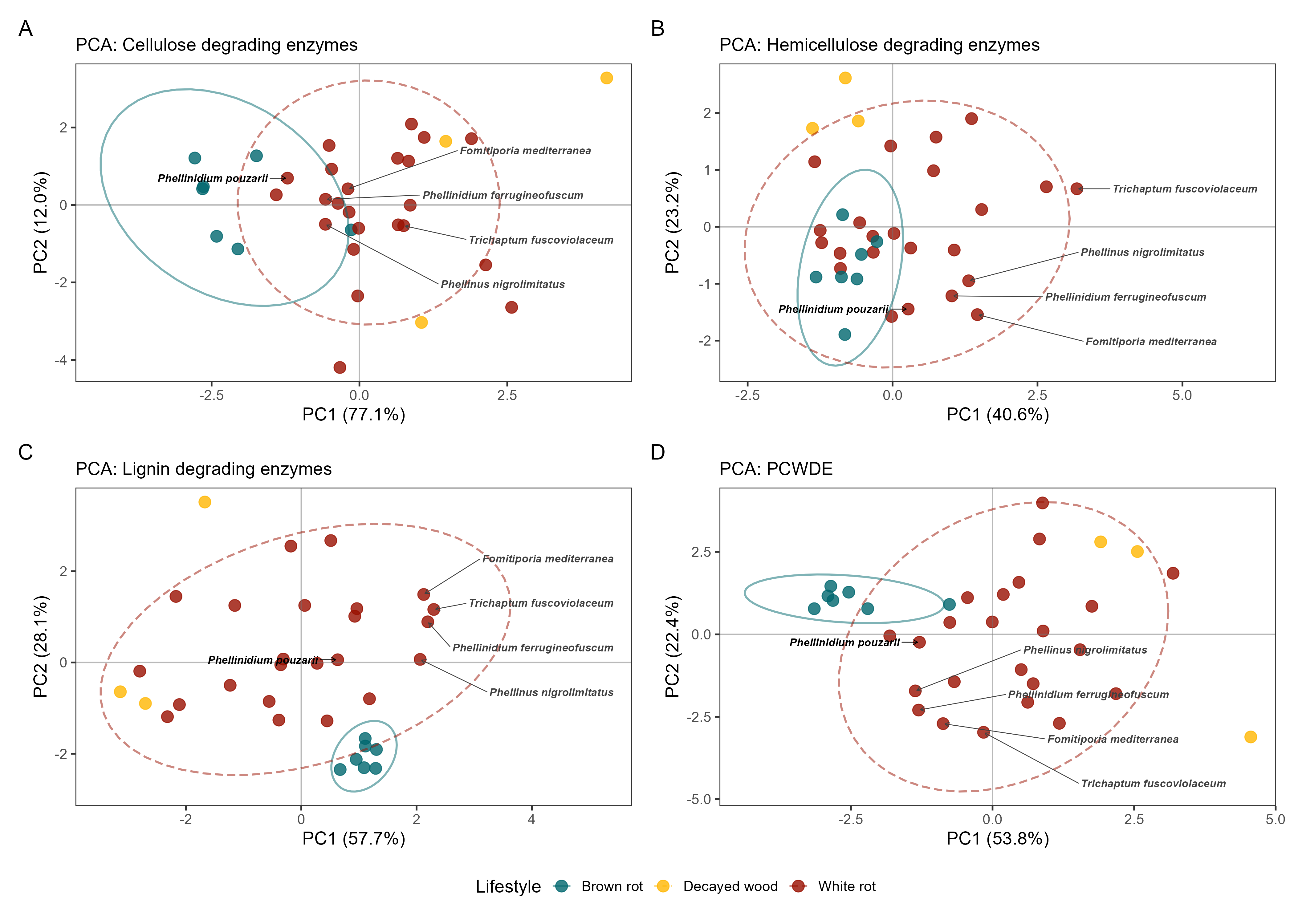


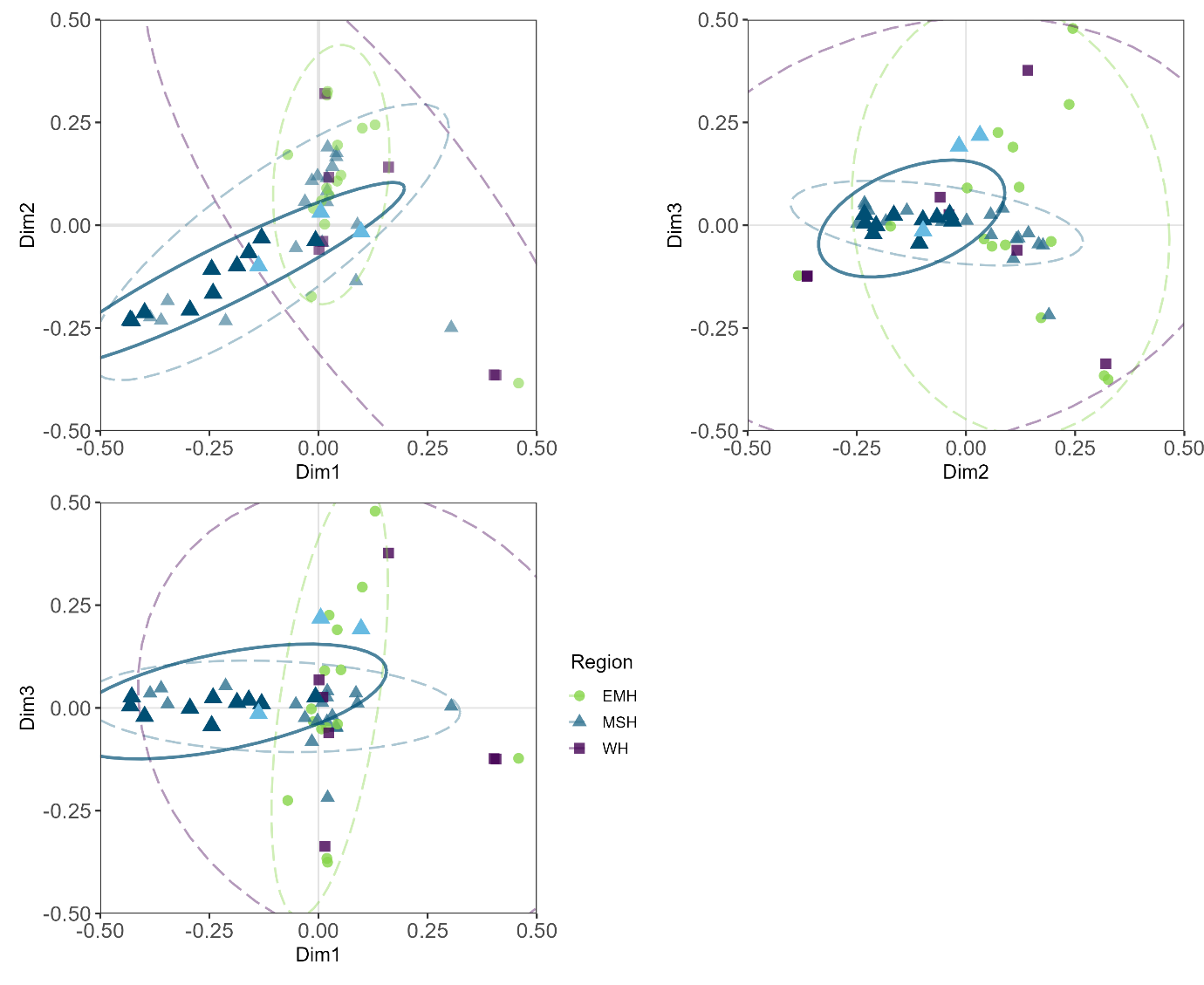


**Figure S3.13:** PCoA of fungal communities from 2022 and 2021 including three logs that were sampled several times (TA1, TA12, TA19). Transparent symbols indicate samples where we did not detect *P. pouzarii* using amplicon sequencing or qPCR (green circle - EMH: Eisenmannhaus, bluegrey triangle - MSH: Mittelsteighütte, purple rectangle -WH: Watzlik-Hain). Solid triangles represent *P. pouzarii* positive samples (exclusively from Mittelsteighütte as we did not detect it anywhere else) from 2021 (dark blue solid triangles) and 2022 (light blue solid triangles). First three axes of the ordination are shown.

## S4: Tables

| **Region/direction** | **Name** | **Sequence (5‘ – 3‘)** | **Citation** |
| --- | --- | --- | --- |
| ITS forward | P7-3N-flTS7 | GTGACTGGAGTTCAGACGTGTGCTCTTCCGATCTNNNGTGARTCATCGAATCTTTG | Ihrmark et al. (2012),  Hendgen et al. (2018) |
|  | P7-4N-flTS7 | GTGACTGGAGTTCAGACGTGTGCTCTTCCGATCTNNNNGTGARTCATCGAATCTTTG |  |
| ITS reverse | P5-2N-ITS4 | ACACTCTTTCCCTACACGACGCTCTTCCGATCTNNNNNTCCTCCGCTTATTGATATGC |  |
|  | P5-1N- ITS4 | ACACTCTTTCCCTACACGACGCTCTTCCGATCTNNNNNNTCCTCCGCTTATTGATATGC |  |
| 16S forward | P5-8N-515F | ACACTCTTTCCCTACACGACGCTCTTCCGATCTNNNNNNNNGTGCCAGCMGCCGCGGTAA | Caporaso et al. (2012) |
|  | P5-7N-515F | ACACTCTTTCCCTACACGACGCTCTTCCGATCTNNNNNNNGTGCCAGCMGCCGCGGTAA |  |
| 16S reverse | P7-2N-806R | GTGACTGGAGTTCAGACGTGTGCTCTTCCGATCTNNGGACTACHVGGGTWTCTAAT |  |
|  | P7-1N-806R | GTGACTGGAGTTCAGACGTGTGCTCTTCCGATCTNGGACTACHVGGGTWTCTAAT |  |
| Illumina index primers | P5 | AATGATACGGCGACCACCGAGATCTACACiiiiiiiiACACTCTTTCCCTACACGACGCTCTTCCGATC*T | Illumina, Inc. San Diego, CA, USA |
|  | P7 | CAAGCAGAAGACGGCATACGAGATiiiiiiiiGTGACTGGAGTTCAGACGTGTGCTCTTCCGATC*T |  |
| *Phellinidium pouzarii*  qPCR primers | PhePou_634  reverse | CCGAAGACGATTAAGAAGCGG | This study |
|  | PhePou_455  forward | TCTTCGCCTTCTTTCGTGGG |  |
|  | PhePou_222  reverse | AAAAACGAAGGAACGCTCGAC |  |
|  | PhePou_43  forward | CTGGTAGCCTCTCTCCGGG |  |

**Table S4.1:** Primers used in this study for metabarcoding and qPCR

**Table S4.2:** Top 20 most abundant fungal OTUs in the 2022 dataset

| **OTU** | **Species** | **Relative abundance (%)** |
| --- | --- | --- |
| OTU_0322 | *Hyphodontia pallidula* | 6.13 |
| OTU_0270 | *Fomitopsis pinicola* | 6.09 |
| OTU_0372 | *Hyphodontia aspera* | 5.24 |
| OTU_0198 | *Ischnoderma benzoinum* | 3.67 |
| OTU_0030 | Unknown basidiomycetous fungus | 3.60 |
| OTU_0284 | *Resinicium bicolor* | 3.43 |
| OTU_0459 | *Basidiodendron trachysporum* | 3.11 |
| OTU_0309 | *Ganoderma applanatum* | 3.00 |
| OTU_0248 | *Heterobasidion* sp. | 2.84 |
| OTU_0155 | *Megacollybia platyphylla* | 2.82 |
| OTU_0214 | *Hericium flagellum* | 2.70 |
| OTU_1261 | *Candida mesenterica* | 2.58 |
| OTU_0140 | *Kneiffiella curvispora* | 2.16 |
| OTU_0235 | *Cystostereum murrayi* | 1.88 |
| OTU_0393 | *Amylostereum chailletii* | 1.73 |
| OTU_0298 | Unknown basidiomycetous fungus | 1.62 |
| OTU_0538 | *Pseudoplectania melaena* | 1.60 |
| OTU_0132 | *Mycena purpureofusca* | 1.58 |
| OTU_0236 | *Neohypochnicium wakefieldiae* | 1.37 |
| OTU_0189 | *Peniophorella pubera* | 1.28 |

**Table S4.3:** Top 20 most abundant bacterial OTUs in the 2022 dataset

| **OTU** | **Genus** | **Relative abundance (%)** |
| --- | --- | --- |
| OTU_00728 | *Methylovirgula******** | 3.43 |
| OTU_00737 | *Conexibacter* | 3.32 |
| OTU_00730 | *Burkholderia-Caballeronia-Paraburkholderia* | 3.22 |
| OTU_00732 | *Roseiarcus* | 2.67 |
| OTU_00740 | *Galbitalea* | 2.59 |
| OTU_00729 | *Bradyrhizobium* | 2.45 |
| OTU_00731 | *Sodalis* | 2.2 |
| OTU_00739 | *Conexibacter* | 2.15 |
| OTU_00735 | WD260 | 1.98 |
| OTU_01354 | *Edaphobacter* | 1.87 |
| OTU_02608 | *Leptothrix* | 1.85 |
| OTU_00743 | *Granulicella* | 1.76 |
| OTU_00742 | *Acidipila-Silvibacterium* | 1.44 |
| OTU_00749 | *Acidisoma* | 1.29 |
| OTU_00781 | *Edaphobacter* | 1.24 |
| OTU_00759 | *Pseudorhodoplanes* | 1.20 |
| OTU_00744 | Unknown *Bacteria* | 1.19 |
| OTU_00758 | *Acidothermus* | 1.14 |
| OTU_00741 | JG36-TzT-191 | 1.05 |

* The genus *Methylovirgula* contains the methylotrophic and acidophilic species *M. ligni* that was reported to be associated with beech wood colonized by the agaric white-rot fungus *Hypholoma fasciculare* (doi:10.1099/ijs.0.010074-0).

**Table S4.4:** PERMANOVA results for fungal and bacterial communities in 2022 (Df: degrees of freedom; italics: p < 0.10, *: p < 0.05, **: p < 0.01, ***: p < 0.001)

|  | **Variable** | **Df** | **Sum of squares** | **R^2^** | **F** | **p** |
| --- | --- | --- | --- | --- | --- | --- |
| **Fungi** | Decay stage | 1 | 0.42 | 0.02 | 0.93 | 0.6 |
|  | Region | 2 | 1.08 | 0.05 | 1.19 | *0.09* |
|  | *P. pouzarii* | 1 | 0.59 | 0.03 | 1.29 | *0.06* |
|  | Residual | 43 | 19.6 | 0.9 |  |  |
|  | Total | 47 | 21.7 | 1.0 |  |  |
| **Bacteria** | Decay stage | 1 | 0.39 | 0.03 | 1.44 | 0.11 |
|  | Region | 2 | 0.70 | 0.05 | 1.27 | 0.12 |
|  | *P. pouzarii* | 1 | 0.22 | 0.02 | 0.82 | 0.60 |
|  | Residual | 43 | 11.8 | 0.89 |  |  |
|  | Total | 47 | 13.07 | 1.00 |  |  |

## References

1. Kradolfer, P., Niederberger, P. & Hütter, R. Tryptophan degradation in Saccharomyces cerevisiae: Characterization of two aromatic aminotransferases. *Arch. Microbiol.* **133**, 242–248 (1982).

2. Iraqui, I., Vissers, S., Cartiaux, M. & Urrestarazu, A. Characterisation of *Saccharomyces cerevisiae* ARO8 and ARO9 genes encoding aromatic aminotransferases I and II reveals a new aminotransferase subfamily. *Mol. Gen. Genet. MGG* **257**, 238–248 (1998).

3. Rząd, K., Milewski, S. & Gabriel, I. Versatility of putative aromatic aminotransferases from *Candida albicans*. *Fungal Genet. Biol.* **110**, 26–37 (2018).

4. Zhuang, N. *et al.* Purification, crystallization and crystallographic analysis of *Dictyostelium discoideum* phenylalanine hydroxylase in complex with dihydrobiopterin and FeIII. *Acta Crystallograph. Sect. F Struct. Biol. Cryst. Commun.* **66**, 463–466 (2010).

5. Zhu, L. *et al.* Cloning, expression and characterization of phenylalanine ammonia-lyase from *Rhodotorula glutinis*. *Biotechnol. Lett.* **35**, 751–756 (2013).

6. Ward, L. C., McCue, H. V. & Carnell, A. J. Carboxyl Methyltransferases: Natural Functions and Potential Applications in Industrial Biotechnology. *ChemCatChem* **13**, 121–128 (2021).

7. Wat, C.-K. & Towers, G. H. N. Phenolic *O*-methyltransferase from *Lentinus lepideus* (basidiomycete). *Phytochemistry* **14**, 663–666 (1975).

8. Birkinshaw, J. H., Bracken, A. & Findlay, W. P. K. Biochemistry of the Wood-rotting Fungi.

9. Liu, Q. *et al.* Rewiring carbon metabolism in yeast for high level production of aromatic chemicals. *Nat. Commun.* **10**, 4976 (2019).

10. Vuralhan, Z. *et al.* Physiological Characterization of the *ARO10* -Dependent, Broad-Substrate-Specificity 2-Oxo Acid Decarboxylase Activity of *Saccharomyces cerevisiae*. *Appl. Environ. Microbiol.* **71**, 3276–3284 (2005).

11. Ehrlich, F. Über die Bedingungen der Fuselölbildung und über ihren Zusammenhang mit dem Eiweißaufbau der Hefe. *Berichte Dtsch. Chem. Ges.* **40**, 1027–1047 (1907).

12. Hazelwood, L. A., Daran, J.-M., van Maris, A. J. A., Pronk, J. T. & Dickinson, J. R. The Ehrlich pathway for fusel alcohol production: a century of research on *Saccharomyces cerevisiae* metabolism. *Appl. Environ. Microbiol.* **74**, 2259–2266 (2008).

13. Dickinson, J. R., Salgado, L. E. J. & Hewlins, M. J. E. The catabolism of amino acids to long chain and complex alcohols in *Saccharomyces cerevisiae*. *J. Biol. Chem.* **278**, 8028–8034 (2003).

14. Yadav, S., Yadav, R. S. S. & Yadav, K. D. S. Stereoselective benzylic hydroxylation of ethylbenzene and propylbenzene using the mycelia of *Aspergillus flavus* MTCC-1783 and MTCC-1884. *Can. J. Chem.* **90**, 597–599 (2012).

15. Shimada, M., Nakatsubo, F., Kirk, T. K. & Higuchi, T. Biosynthesis of the secondary metabolite veratryl alcohol in relation to lignin degradation in *Phanerochaete chrysosporium*. *Arch. Microbiol.* **129**, 321–324 (1981).

16. Uzura, A., Suzuki, T., Katsuragi, T. & Tani, Y. Involvement of cytochrome P450 in hydroxylation of propylbenzene by *Fusarium moniliforme* strain MS31. *J. Biosci. Bioeng.* **92**, 580–584 (2001).

17. Kluge, M., Ullrich, R., Scheibner, K. & Hofrichter, M. Stereoselective benzylic hydroxylation of alkylbenzenes and epoxidation of styrene derivatives catalyzed by the peroxygenase of *Agrocybe aegerita*. *Green Chem.* **14**, 440–446 (2012).

18. Churakova, E. *et al.* Specific Photobiocatalytic Oxyfunctionalization Reactions<a/>. *Angew. Chem.* **45**, 10904–10907 (2011).

19. Clausen, M., Lamb, C. J., Megnet, R. & Doerner, P. W. PAD1 encodes phenylacrylic acid decarboxylase which confers resistance to cinnamic acid in *Saccharomyces cerevisiae*. *Gene* **142**, 107–112 (1994).

20. Richard, P., Viljanen, K. & Penttilä, M. Overexpression of PAD1 and FDC1 results in significant cinnamic acid decarboxylase activity in *Saccharomyces cerevisiae*. *AMB Express* **5**, 12 (2015).

21. Stratford, M., Plumridge, A. & Archer, D. B. Decarboxylation of Sorbic Acid by Spoilage Yeasts Is Associated with the PAD1 Gene. *Appl. Environ. Microbiol.* **73**, 6534–6542 (2007).

22. Plumridge, A. *et al.* The decarboxylation of the weak-acid preservative, sorbic acid, is encoded by linked genes in *Aspergillus* spp. *Fungal Genet. Biol. FG B* **47**, 683–692 (2010).

23. Tischler, D. *et al.* StyA1 and StyA2B from *Rhodococcus opacus* 1CP: a Multifunctional Styrene Monooxygenase System. *J. Bacteriol.* **192**, 5220–5227 (2010).

24. Cox, H. H. *et al.* Styrene metabolism in *Exophiala jeanselmei* and involvement of a cytochrome P-450-dependent styrene monooxygenase. *Appl. Environ. Microbiol.* **62**, 1471–1474 (1996).

25. Smit, M. S. Fungal epoxide hydrolases: new landmarks in sequence-activity space. *Trends Biotechnol.* **22**, 123–129 (2004).

26. McKenna, R., Pugh, S., Thompson, B. & Nielsen, D. R. Microbial production of the aromatic building-blocks (S)-styrene oxide and (R)-1,2-phenylethanediol from renewable resources. *Biotechnol. J.* **8**, 1465–1475 (2013).

27. Bernardo, A., Burgos, J. & Martín, R. Purification and some properties of L-glycol dehydrogenase from hen’s muscle. *Biochim. Biophys. Acta* **659**, 189–198 (1981).

28. Janssen, F. W. & Ruelius, H. W. Alcohol oxidase, a flavoprotein from several basidiomycetes species: Crystallization by fractional precipitation with polyethylene glycol. *Biochim. Biophys. Acta BBA - Enzymol.* **151**, 330–342 (1968).

29. Gutiérrez-Corona, J. F. *et al.* Fungal Alcohol Dehydrogenases: Physiological Function, Molecular Properties, Regulation of Their Production, and Biotechnological Potential. *Cells* **12**, 2239 (2023).

30. Westrick, N. M., Park, S. C., Keller, N. P., Smith, D. L. & Kabbage, M. A broadly conserved fungal alcohol oxidase (AOX) facilitates fungal invasion of plants. *Mol. Plant Pathol.* **24**, 28–43 (2023).

31. Durham, D. R. Initial reactions involved in the dissimilation of mandelate by *Rhodotorula graminis*. *J. Bacteriol.* **160**, 778–780 (1984).

32. Rao, D. N. R. & Vaidyanathan, C. S. Metabolism of mandelic acid by *Neurospora crassa*. *Can. J. Microbiol.* **23**, 1496–1499 (1977).

33. Ullrich, R. & Hofrichter, M. The haloperoxidase of the agaric fungus *Agrocybe aegerita* hydroxylates toluene and naphthalene. *FEBS Lett.* **579**, 6247–6250 (2005).

34. Faber, B. W., van Gorcom, R. F. & Duine, J. A. Purification and characterization of benzoate-para-hydroxylase, a cytochrome P450 (CYP53A1), from *Aspergillus niger*. *Arch. Biochem. Biophys.* **394**, 245–254 (2001).

35. Shang, Y., Wei, W., Zhang, P. & Ye, B.-C. Engineering *Yarrowia lipolytica* for Enhanced Production of Arbutin. *J. Agric. Food Chem.* **68**, 1364–1372 (2020).

36. Floudas, D. *et al.* The Paleozoic Origin of Enzymatic Lignin Decomposition Reconstructed from 31 Fungal Genomes. *Science* **336**, 1715–1719 (2012).

37. Zhao, H. *et al.* Insights into the Ecological Diversification of the Hymenochaetales based on Comparative Genomics and Phylogenomics With an Emphasis on *Coltricia*. *Genome Biol. Evol.* **15**, evad136 (2023).

38. Sønstebø, J. H. *et al.* Population genomics of a forest fungus reveals high gene flow and climate adaptation signatures. *Mol. Ecol.* **31**, 1963–1979 (2022).
